# A linkage map of transcribed single nucleotide polymorphisms in rohu (*Labeo rohita*) and QTL associated with resistance to *Aeromonas hydrophila*

**DOI:** 10.1186/1471-2164-15-541

**Published:** 2014-06-30

**Authors:** Nicholas Robinson, Matthew Baranski, Kanta Das Mahapatra, Jatindra Nath Saha, Sweta Das, Jashobanta Mishra, Paramananda Das, Matthew Kent, Mariann Arnyasi, Pramoda Kumar Sahoo

**Affiliations:** 1 Breeding and Genetics, Nofima, PO Box 5010, 1432 Ås, Norway; 2 Central Institute of Freshwater Aquaculture, Kausalyaganga, Bhubaneswar, India; 3 Biological Sciences, Flinders University, Bedford Park, Australia; 4 Centre for Integrative Genetics, University of Life Sciences, Ås, Norway

**Keywords:** *Labeo rohita*, Single nucleotide polymorphism, Transcribed sequences, Linkage map, Quantitative trait loci, *Aeromonas hydrophila* resistance

## Abstract

**Background:**

Production of carp dominates world aquaculture. More than 1.1 million tonnes of rohu carp, *Labeo rohita* (Hamilton), were produced in 2010. *Aeromonas hydrophila* is a bacterial pathogen causing aeromoniasis in rohu, and is a major problem for carp production worldwide. There is a need to better understand the genetic mechanisms affecting resistance to this disease, and to develop tools that can be used with selective breeding to improve resistance. Here we use a 6 K SNP array to genotype 21 full-sibling families of *L. rohita* that were experimentally challenged intra-peritoneally with a virulent strain of *A. hydrophila* to scan the genome for quantitative trait loci associated with disease resistance.

**Results:**

In all, 3193 SNPs were found to be informative and were used to create a linkage map and to scan for QTL affecting resistance to *A. hydrophila*. The linkage map consisted of 25 linkage groups, corresponding to the number of haploid chromosomes in *L. rohita*. Male and female linkage maps were similar in terms of order, coverage (1384 and 1393 cM, respectively) and average interval distances (1.32 and 1.35 cM, respectively). Forty-one percent of the SNPs were annotated with gene identity using BLAST (cut off E-score of 0.001). Twenty-one SNPs mapping to ten linkage groups showed significant associations with the traits *hours of survival* and *dead or alive* (*P* <0.05 after Bonferroni correction). Of the SNPs showing significant or suggestive associations with the traits, several were homologous to genes of known immune function or were in close linkage to such genes. Genes of interest included heat shock proteins (70, 60, 105 and “small heat shock proteins”), mucin (5b precursor and 2), lectin (receptor and CD22), tributyltin-binding protein, major histocompatibility loci (I and II), complement protein component c7-1, perforin 1, ubiquitin (ligase, factor e4b isoform 2 and conjugation enzyme e2 c), proteasome subunit, T-cell antigen receptor and lymphocyte specific protein tyrosine kinase.

**Conclusions:**

A panel of markers has been identified that will be validated for use with both genomic and marker-assisted selection to improve resistance of *L. rohita* to *A. hydrophila*.

## Background

Carp is one of the world’s most important group of aquaculture species, with production of Rohu carp (*Labeo rohita* Hamilton) accounting for around 1.2 million tonnes in 2010 [1]. Production occurs in India, Bangladesh, Myanmar, Laos and Thailand and most of the fish is consumed within these countries. A selective breeding program established by the Central Institute of Freshwater Aquaculture in Bhubaneswar India has a focus on increasing the growth rate of the fish and has been supplying a genetically improved variety of *L. rohita* called *Jayanti* rohu to the farmers and hatcheries of various States in India since 1992. A 17% percent higher average growth rate per generation was achieved after 4 generations of selective breeding [2] and eight generations of selection have now been completed with a similar selection response. Rohu is efficiently grown in earthen ponds, however disease prevention in this environment is difficult, and mortality and growth loss from disease in India is high.

*Aeromonas hydrophila* is an endemic motile pathogenic bacteria causing haemorrhaging and ulceration when fish are stressed as reviewed by [3]. *A. hydrophila* is widespread and difficult to control and treat as there are no effective drugs or vaccines. The disease *aeromoniasis* caused by *A. hydrophila* infection is a world-wide problem affecting many fish species. Significant additive genetic variation affecting the survival of rohu exposed to experimental challenge tests with *A. hydrophila* has been found [4]; however, rohu is not an ideal model species for studying the genetics of disease resistance. Mortalities occur quickly (sometimes within 30 hours after experimental challenge, [4]) and differences in the challenge infection procedure are believed to affect expression of the genetic potential to survive this disease. Even so, one generation of divergent selection based on challenge test data has been shown to result in significantly higher average rates of survival (73.3 ± 3.3% versus 16.7 ± 3.3%), blood phagocyte respiratory burst activity, serum myeloperoxidase activity and ceruloplasmin level in resistant compared to susceptible line rohu [5]. A major limitation to selective breeding is the inability to directly test highly valuable broodstock by challenging them to the disease.

Knowledge about causative genes, or markers associated with genes affecting disease resistance, could be used to increase the rate of genetic improvement through selective breeding. Markers for disease resistance have been detected and applied to the selective breeding of other teleost species [6-8], but little knowledge exists for *L. rohita*, and resources needed to develop such tests (eg. linkage maps for polymorphic markers) have been lacking. RNA-sequencing has recently been performed to characterise the transcriptomes of selected lines of *L. rohita*, and to concurrently identify SNPs and indels in transcribed genes [9]. Quantitative analysis of RNA-seq data revealed that lines of rohu selected for resistance to *A. hydrophila* showed higher fold naïve expression and allele frequency differences for a number of genes with putative functions affecting immune response when compared to lines selected for susceptibility to *A. hydrophila*. These genes included major histocompatibility class I loci, heat shock proteins 30, 70 and 90, glycoproteins, serum lectin and galactoside-binding soluble lectin. Ceruloplasmin is 4.58 times more highly expressed in resistant than in susceptible line rohu carp that were selected based on family challenge test survival to *A. hydrophila*[10]. SNP polymorphisms at superoxide dismutase 3, an antioxidant enzyme, has also been found to be associated with resistance to *A. hydrophila* in the freshwater mussel *Hyriopsis cumingii*[11].

Here we genotype full-sibling families using an Illumina iSelect array containing SNPs found in transcribed genes, in order to produce a genetic linkage map of the *L. rohita* genome and simultaneously scan the genome of challenge tested families for variation associated with resistance to *A. hydrophila*.

## Results

### Linkage map

A conversion rate of 87.2% meant that the SNP-array used in this study contained 5,232 of the original 6,000 assays (within the manufacturers specified tolerances). After automatic and manual clustering, 3242 markers (62%) fell into the usable “SNP” marker category, with the remainder being fails, monomorphic or low call-confidence markers. Approximately 2% of markers did not segregate according to Mendelian expectations in some of the 21 families genotyped (*P* <0.05, after Bonferroni correction).

In total, 3193 informative SNP markers mapped to 25 linkage groups (Additional files 1 and 2). The female and male maps contained 3008 and 3071 SNP markers respectively and 2886 SNP markers were informative for both maps.

The female linkage map covered 1384 cM with an average interval of 1.32 cM and a maximum interval of 12.7 cM (Table 1). The length of the 25 linkage groups ranged from 45.4 to 75.9 cM and the number of markers varied from 83 to 216 per group. The genome length estimate for the female was 1407 cM resulting in coverage of 99% of the genome within 1 cM of a framework marker.

**Table 1:** **Comparison of map intervals between male and female *L. rohita *** **linkage maps**

		**Maximum distance (cM)**		**Total distance (cM)**	
**LG**	**SNPs**	**Female**	**Male**	**Female**	**Male**
1	126	3.7	2.2	51	55.3
2	100	8.6	3.5	58.1	40.9
3	105	5.7	3.8	55.4	51.6
4	140	4.5	3.8	53	57.9
5	152	4.4	2.9	54.5	49.2
6	120	12.7	8.5	75.9	52.4
7	234	4	2.5	58.5	58.3
8	123	11.1	4.8	62	58.4
9	124	2.5	3.7	49.6	53.4
10	130	3.1	6.2	45.4	50.4
11	116	5.8	5.5	49	57.4
12	100	4.8	23.6	60.9	71.5
13	89	8.8	3.4	60.1	34.2
14	101	4	37.1	46.2	87.6
15	158	5.2	3.4	49.7	63.4
16	96	5.5	3.8	57.2	53.7
17	163	3.8	3.6	66.5	48.8
18	139	6.8	5.7	55.7	62.9
19	113	7.5	3.6	52.4	55.6
20	148	5.2	7.2	48.3	59.3
21	135	4.1	2.5	48.6	56.4
22	152	4.8	3	54.3	44.4
23	122	5.2	7	58.6	54.2
24	103	10	10	60.4	58.5
25	104	7.6	4.7	52.7	57.8

LG, linkage group; SNPs, -number of markers on sex-average map.

The male linkage map covered 1393.5 cM with an average interval of 1.35 cM and a maximum interval of 37.1 cM (Table 1). The length of the linkage groups ranged from 34.2 to 87.6 cM and the number of markers varied from 87 to 220 per group. The genome length estimate for the male was 1416 cM resulting in coverage of 99% of the genome within 1 cM of a framework marker.

Little difference was detected between the total lengths and map distances between the male and female specific maps (Table 1). The male map was 9 cM longer than the female map, 122 female informative markers were linked in the female map but unlinked in the male map, while 185 male informative markers were linked on the male map but not the female map.

Overall, *L. rohita* linkage groups 1–25 corresponded with *D. rerio* chromosome numbers 15, 24, 17, 16, 19, 21, 3, 1, 13, 23, 12, 9, 25, 4, 2, 11, 7, 8, 14, 22, 6, 5, 20, 10 and 18 respectively. There was strong correspondence between the order of genes within linkage groups for *L. rohita* and the order of the same genes within chromosomes in *Danio rerio* (zebra fish, Additional file 3) although some differences in the ordering of blocks of genes within *L. rohita* linkage groups, compared to *D. rerio* chromosomes, were observed. For instance, the gene order from 0 – 47 cM of LG18 in *L. rohita* corresponds to much the same order as from 55,178,337 bp – 589,315 bp of chromosome 8 in *D. rerio*, except that the block of genes between positions 2,781,136 and 8,043,460 bp on chromosome 8 in *D. rerio* run from 48.9 – 56.6 cM in *L. rohita* linkage group 18, indicating that there has been a rearrangement at the end of this linkage group/chromosome. The similarity between *L. rohita* and *D. rerio* gene sequences was on average 87% (±0.3% SE, average *L. rohita* transcript length of 155 bp). Forty-one percent of the mapped *L. rohita* SNPs were annotated with gene identity using BLAST (cut off E-score of 0.001).

### Challenge tests

The most susceptible and resistant 20% of animals from each of the challenge tested families were sampled for DNA extraction and a random set of these selected for genotyping giving an overall mean survival 12.19 ± 9.89 SD hours post *A. hydrophila* challenge. The spread of hours survival ranged from 2 to 26 hours. Plots of hours survival for the animals genotyped within each of the 21 full-sibling family groups that were challenged and sampled are shown in Additional file 4: Figure S1.

### Genetic parameters associated with *A. hydrophila* resistance

Significant effects of tank and pedigree on *hours of survival* (Table 2) and *dead or alive* traits (not shown) were detected. Heritability of *hours of survival* and *dead or alive* traits were low (0.05 and 0.07 respectively) but significant (0.02 - 0.16 and 0.02 – 0.20 lower and upper 95% confidence intervals for the two traits respectively). The genetic correlation between the *hours of survival* and *dead or alive* traits (0.79) was positive, high and significant (0.59 and 0.94 lower and upper 95% confidence intervals respectively).

**Table 2:** **MCMCglmm analysis under an animal model of *hours of survival *** **after experimental challenge to *A. hydrophila***

**95% confidence limits**					
**Parameter**	**Mean**	**Lower**			**Upper**	**Effective sample**	***P*-value**
Genetic structure					
*animal*	7.2	1.7			15.3	1220	
*ID*	24.9	1.1			66.9	359	
*units*	60.9	16.1			89.5	369.2	
(Intercept)	0.1	-2.9			2.9	1519	0.961
*tank*	5.6	3.3			7.7	1400	<7e-04***
*ped2008F12*	7.3	3.3			11	1613	<7e-04***
*ped2008F13*	4.7	-0.2			9.4	1400	0.056
*ped2008F21*	7.6	3.7			11.0	1400	<7e-04***
*ped2008F22*	7.9	4.4			12	1400	<7e-04***
*ped2008F24*	-4.6	-9.1			0.8	1400	0.067
*ped2008F29*	0.9	-2.7			5	1400	0.63
*ped2008F34*	-2.7	-9.1			3.2	1400	0.379
*ped2008F38*	-8.5	-16.7			-0.8	1400	0.043
*ped2008F41*	12.2	8.2			16.1	1400	<7e-04***
*ped2008F44*	-1.9	-20.1			16.5	1400	0.826
*ped2008F49*	1.4	-3.4			6.0	1400	0.553

Tank and family (ped2008F12 – ped2008F49) were fitted as fixed effects. Mean, mean of posterior distribution. *, *P* < 0.05. **, *P* <0.01. ***, *P* <0.001.

### Quantitative trait loci (QTL) associated with resistance to *A. hydrophila*

The quality control steps excluded all markers whose inheritance was non-Mendelian and all individuals who could be excluded with parentage analysis, leaving 3193 markers and 965 phenotyped and genotyped progeny of 21 sires and 21 dams for association analysis. Although tank and pedigree (family) were found to be significant fixed effects, their inclusion in the model for QTL analysis did not affect the SNPs found to be associated with either trait or the overall level of significance for the associations. Results for the simplest model (without fixed effects) are therefore presented here.

Half-sib regression interval mapping analysis detected one genome-wide significant QTL for hours of survival on LG15 (dam-based) and five suggestive QTL both hours of survival and the binary dead/alive trait (Table 3). In only one case was a suggestive QTL detected for both traits on the same linkage group (LG3). In all cases we were able to infer that two of the seven analysed parents were segregating for the QTL.

**Table 3:** Summary of suggestive and significant QTL detected using GridQTL half-sib regression analysis

**LG**	**Analysis (Sire/Dam)**	**Trait**	**Pos**	**F-stat**	**Segregation**
3	Dam	Dead/alive	22 cM	3.39*	(A & C)
3	Dam	Hours	22 cM	3.57*	(A & C)
7	Sire	Dead/alive	2 cM	3.42*	(A & D)
14	Dam	Dead/alive	39 cM	3.26*	(A & E)
15	Dam	Hours	29 cM	4.68**	(C & G)
19	Dam	Dead/alive	13 cM	2.81*	(A & C)
23	Sire	Hours	0 cM	3.23*	(C & D)

*Chromosome-wide significance.

**Genome-wide significance.

LG, linkage group. Pos, position on LG in cM. Segregation, families showing segregation for the QTL.

**Figure 1:**
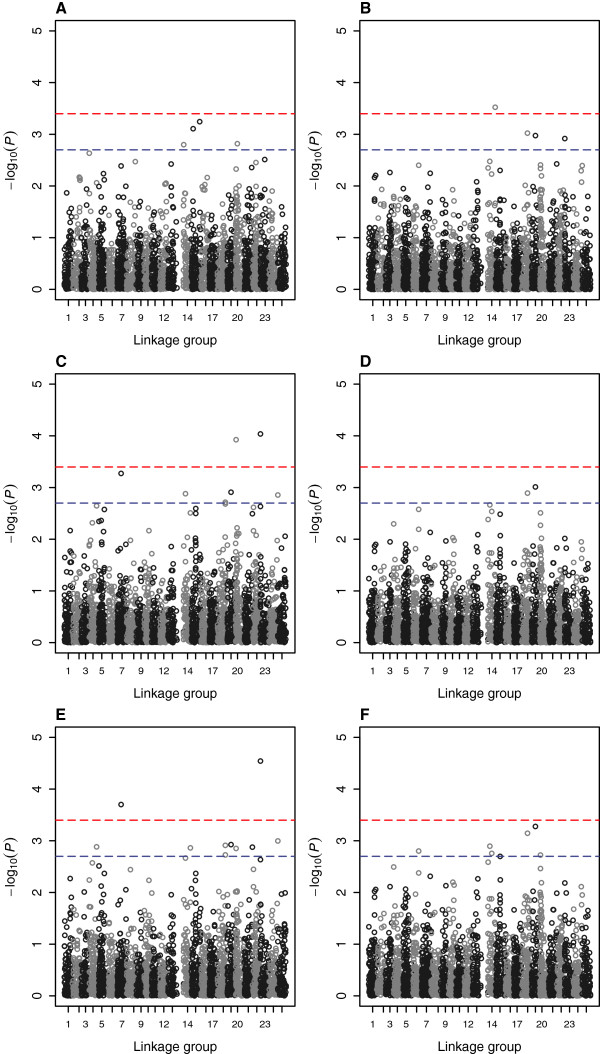
**Manhattan plots showing corrected *P*-values with 1 degrees of freedom after permutation testing for SNPs across the 25 linkage groups for traits *hours of survival *(plots A, C and E) and *dead or alive *(plots B, D and F) for tests QFAM (plot A), ASSOC (plot B), FASTA (plots C and D) and GRAMMA (plots E and F).** Linkage group positions are shown in centimorgons (cM) on the horizontal axis. Upper and lower dotted lines mark significance thresholds after Bonferroni correction of *P* <0.01 and *P* <0.05 respectively.

**Figure 2:**
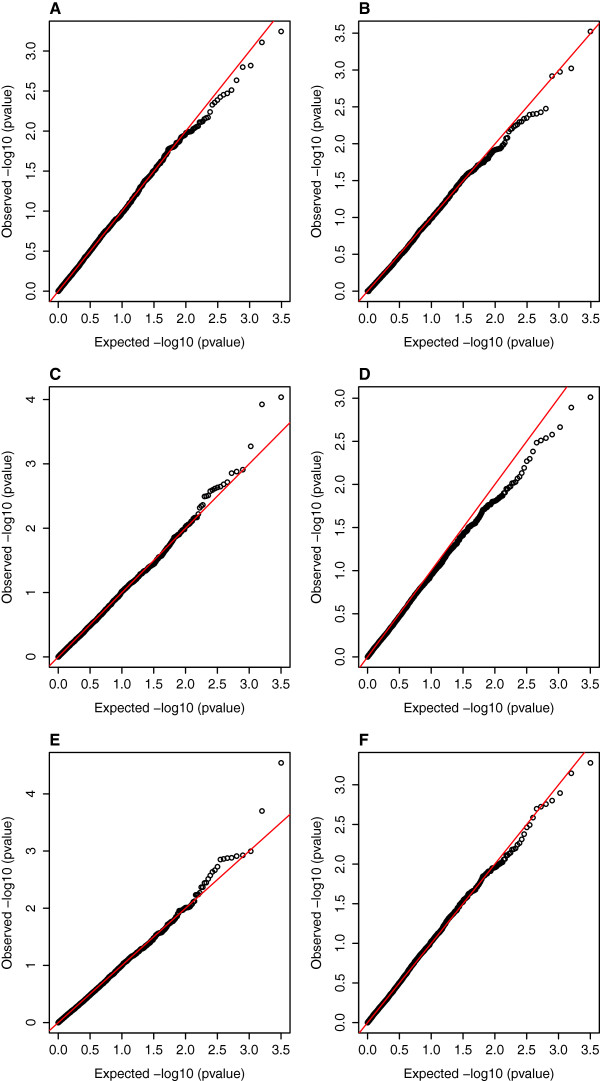
**QQ plots showing observed and expected corrected *P*-values with 1 degrees of freedom after permutation for SNPs tested across the 25 linkage groups for *traits hours of survival *** **(plots A, C and E) and *dead or alive *** **(plots B, D and F) for tests QFAM (plot A), ASSOC (plot B), FASTA (plots C and D) and GRAMMA (plots E and F).**

**Figure 3:**
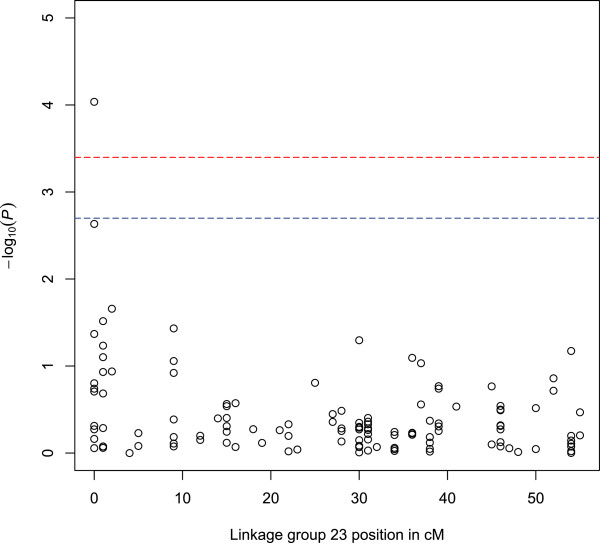
**Manhattan plot showing corrected *P*-values with 1 degrees of freedom after permutation testing for SNPs across linkage group 23 for the trait *hours of survival *for test FASTA.** Linkage group positions are shown in centimorgons (cM) on the horizontal axis. Upper and lower dotted lines mark significance thresholds after Bonferroni correction of *P* <0.01 and *P* <0.05 respectively.

The genome-wide association studies (GWAS) detected many regions with suggestive QTL for *A. hydrophila* resistance for the two traits (*P* <0.001 before Bonferroni correction, Tables 4 and 5). Twenty-one SNPs mapping to ten linkage groups (4, 7, 14, 15, 18–21, 23, 24), and covering possibly twelve distinct regions in total, showed significant associations with the trait *hours of survival* (*P* <0.05 after Bonferroni correction, Figures 1A, C and E and 2A, C and E). Of these, SNPs mapping to linkage groups 7, 20 and 23 were significant at *P* <0.01 level after Bonferroni correction for some tests and one SNP mapping to 0 cM on linkage group 23 (93296_256 with homology to loc795887 uncharacterised protein from *D. rerio*) was significant at *P* <0.001 after Bonferroni correction for the GRAMMA test (Figure 3). Linkage group 23 corresponds to chromosome 20 of the *D. rerio* genome (Additional file 3). Genes of potential interest in terms of immune function mapping to this region of LG23 included dermatin sulphate epimerase (SNP 55156_84, Additional file 1).

**Table 4:** **Suggestive and significant QTL for trait *hours of survival *after challenge with *A. hydrophila *detected using PLINK (QFAM) and GenAbel (FASTA and GRAMMA) analyses in 21** ***L. rohita *families**

**LG**	**Pos**	**SNP**	**Test**	**N**	**Effect**	**Stat**	***P *-value**	**Sig**	**GeneID**
1	37.3	55086_181	FASTA	963	-2.22(0.82)	7.32	0.0068		Small heat shock
1	37.3	55086_181	GRAMMA	963	-1.18 (0.56)	4.47	0.00538		Small heat shock
2	43.9	121615_93	QFAM	1021		-1.449	0.00675		
2	47.4	6937_74	QFAM	1022		1.48	0.00772		
2	48.4	61599_86	QFAM	1022		1.48	0.00708		
2	48.4	83153_20	QFAM	1022		1.48	0.00775		
3	20.3	68284_87	GRAMMA	979	-1.12 (0.57)	3.85	0.00979		
4	0	84080_123	GRAMMA	978	1.05 (0.5)	4.4	0.00575		
4	0	84080_123	QFAM	1021		1.708	0.00232		
4	19.7	65405_244	FASTA	979	-2.07(0.73)	7.95	0.00482		
4	19.7	65405_244	GRAMMA	979	-1.26 (0.55)	5.21	0.00268		
4	46.8	110358_408	FASTA	979	2.18(0.71)	9.33	0.00226		c-type natriuretic peptide 1 precursor
4	46.8	110358_408	GRAMMA	979	1.3 (0.53)	5.96	0.00131	*	c-type natriuretic peptide 1 precursor
4	46.8	110358_408	QFAM	1022		2.058	0.00867		c-type natriuretic peptide 1 precursor
5	9.6	4797_109	FASTA	978	-1.57(0.55)	8.06	0.00452		
5	9.6	4797_109	GRAMMA	978	-0.92 (0.41)	5.06	0.00307		
5	23.8	83820_94	FASTA	979	-2.05(0.72)	8.14	0.00434		Brain specific kinase 146
5	23.8	83820_94	GRAMMA	979	-0.97 (0.48)	4.12	0.00753		Brain specific kinase 146
5	38.3	1886_343	FASTA	979	1.71(0.64)	7.24	0.00712		loc100037090 protein
5	38.3	1886_343	QFAM	1022		1.826	0.00577		loc100037090 protein
5	43.4	53540_630	FASTA	976	2.76(0.92)	9.03	0.00265		protein phosphatase regulatory subunit 10
5	43.4	53540_630	GRAMMA	976	1.28 (0.59)	4.71	0.00429		Protein phosphatase Regulatory subunit 10
5	43.4	75322_75	QFAM	1022		-1.517	0.00759		
7	23.4	62374_157	FASTA	968	-3.59(1.04)	11.99	0.00053	*	
7	23.4	62374_157	GRAMMA	968	-2.26 (0.8)	7.99	0.0002	**	
7	23.4	62374_157	QFAM	1016		-4.763	0.0041		
7	53.7	87974_385	GRAMMA	979	-0.95 (0.48)	3.96	0.00886		sec14-like 1 (cerevisiae)
8	23.3	136056_232	GRAMMA	979	-1.04 (0.47)	4.89	0.00362		
8	54.9	31265_40	QFAM	1022		1.369	0.00338		cd22 antigen
10	29.6	82862_249	FASTA	979	-1.99(0.73)	7.32	0.00683		
10	29.6	82862_249	GRAMMA	979	-1.11 (0.53)	4.39	0.00582		
12	31.1	57295_319	QFAM	1022		-1.117	0.00924		
12	37.8	52515_4545	QFAM	1022		1.667	0.0088		titin a
12	37.8	63497_114	QFAM	1022		1.667	0.009		Unnamed protein product [Tetraodon nigroviridis]
13	19.8	64435_287	QFAM	1022		1.813	0.00376		Proline rich 5
14	1.9	132996_241	QFAM	1021		1.646	0.00159	*	
14	12.5	112228_90	FASTA	979	2.09(0.65)	10.32	0.00132	*	
14	12.5	112228_90	GRAMMA	979	1.02 (0.44)	5.43	0.00216		
14	24.1	96692_167	FASTA	979	1.53(0.59)	6.79	0.00918		Novel protein prickle-like family
14	43.9	113696_50	FASTA	976	-3.39(1.15)	8.75	0.0031		Perforin 1 (pore forming protein)
14	43.9	113696_50	GRAMMA	976	-2.22 (0.91)	5.92	0.00137	*	Perforin 1 (pore forming protein)
15	1.1	13736_229	GRAMMA	979	-1 (0.5)	4.01	0.00844		
15	12.5	55466_266	QFAM	1022		1.383	0.00078	*	sept2 protein
15	26.7	110434_333	FASTA	979	-1.93(0.75)	6.72	0.00956		t cell antigen receptor alpha chain c region
15	26.7	110434_333	GRAMMA	979	-1.18 (0.56)	4.39	0.00584		t cell antigen receptor alpha chain c region
15	28.9	54100_91	FASTA	979	2.06(0.68)	9.11	0.00255		cerebellin 1 precursor
15	28.9	60130_224	FASTA	978	-1.95(0.66)	8.7	0.00318		
15	28.9	60130_224	GRAMMA	978	-0.99 (0.46)	4.71	0.00429		
15	54.4	94824_55	QFAM	1022		-1.559	0.00057	*	Adaptor-related protein complex mu 1 subunit
16	23.8	115737_104	QFAM	1020		-1.206	0.0094		mucin-5b precursor (mucin 5 subtype tracheobronchial) (high molecular weight salivary mucin mg1) (sublingual gland mucin)
16	39.2	136477_76	QFAM	1022		2.121	0.00687		sp2 transcription factor
18	8.4	60684_109	GRAMMA	978	-0.89 (0.45)	3.88	0.00958		
18	48.9	52766_1600	FASTA	979	-1.8(0.69)	6.81	0.00909		solute carrier family 25 (mitochondrial carrier phosphate carrier) member 25
18	49.9	116665_768	FASTA	979	1.95(0.63)	9.62	0.00193	*	ptc7 protein phosphatase homolog (cerevisiae)
18	49.9	116665_768	GRAMMA	979	1.14 (0.46)	6.04	0.00122	*	ptc7 protein phosphatase homolog (cerevisiae)
18	49.9	13427_160	FASTA	979	2.06(0.67)	9.48	0.00208		
18	49.9	13427_160	GRAMMA	979	1.13 (0.48)	5.58	0.00188	*	
19	23.8	111569_63	FASTA	979	2.49(0.77)	10.44	0.00123	*	tbt-binding protein
19	23.8	111569_63	GRAMMA	979	1.35 (0.55)	6.07	0.00119	*	tbt-binding protein
20	0.8	54931_324	GRAMMA	979	1.16 (0.59)	3.86	0.0097		solute carrier family member 34
20	3.3	134730_80	FASTA	970	-2.64(0.69)	14.81	0.00012	**	
20	3.3	134730_80	GRAMMA	970	-0.99 (0.41)	5.89	0.00141	*	
20	3.3	134730_80	QFAM	1013		-2.504	0.0047		
20	7.9	88771_72	FASTA	954	-1.53(0.58)	6.89	0.00866		
20	9.4	103839_124	FASTA	970	2.22(0.81)	7.54	0.00603		
20	9.4	103839_124	GRAMMA	970	1.07 (0.54)	3.86	0.00974		
20	9.4	110140_1196	FASTA	979	1.6(0.6)	7.06	0.00787		glycerol-3-phosphate dehydrogenase
20	9.4	110140_1196	GRAMMA	979	0.82 (0.42)	3.9	0.00938		glycerol-3-phosphate dehydrogenase
20	11.1	59816_21	QFAM	1022		-1.607	0.00152	*	novel protein vertebrate stabilin 2
20	20.3	134434_222	FASTA	978	-2.03(0.76)	7.09	0.00776		
20	21.6	20086_69	QFAM	1022		1.556	0.00996		vacuolar protein sorting 4b
21	47.1	111636_59	QFAM	1022		1.417	0.0044		kiaa1219 protein
21	51.1	54579_132	FASTA	979	1.63(0.55)	8.68	0.00321		
21	51.1	54579_132	GRAMMA	979	1.04 (0.43)	5.95	0.00133	*	
22	6	88524_52	FASTA	979	1.55(0.57)	7.31	0.00687		
22	6	88524_52	GRAMMA	979	0.97 (0.44)	4.9	0.00357		
22	6.4	94914_114	FASTA	979	2.07(0.68)	9.2	0.00242		
22	6.4	94914_114	GRAMMA	979	0.86 (0.42)	4.09	0.00775		
22	22.3	58881_141	QFAM	1022		-1.781	0.00352		tuberous sclerosis 1
22	23.2	554_399	FASTA	979	-1.89(0.72)	6.94	0.00841		dipeptidyl peptidase 7
22	23.2	554_399	GRAMMA	979	-0.98 (0.5)	3.86	0.00974		dipeptidyl peptidase 7
23	0	93296_256	FASTA	978	3.71(0.95)	15.3	0.00009	**	loc795887 protein
23	0	93296_256	GRAMMA	978	2.28 (0.72)	10.11	0.00003	***	loc795887 protein
23	0.9	110253_351	FASTA	979	2.35(0.77)	9.27	0.00232		novel protein
23	0.9	110253_351	GRAMMA	979	1.27 (0.55)	5.36	0.00232		novel protein
23	27.4	64731_210	QFAM	1022		1.41	0.00308		
24	55.9	67606_298	FASTA	979	-3.63(1.14)	10.21	0.0014	*	serine long chain base subunit 1
24	55.9	67606_298	GRAMMA	979	-2.07 (0.83)	6.25	0.00101	*	serine long chain base subunit 1
25	46.1	54056_576	FASTA	979	1.7(0.65)	6.87	0.00876		n-ethylmaleimide sensitive fusion protein attachment protein alpha

LG, linkage group; Pos, location on LG in centimorgans; N, number of progeny and parents analysed; Effect, allele substitution effect of the minor allele with standard error in parenthesis (FASTA and GRAMMAS); Stat, test statistic linear regression coefficient for QFAM, chi-square with one degree of freedom for FASTA and GRAMMA analyses; P, point-wise empirical *P*-value (QFAM) or permuted *P*-value with one degree of freedom corrected for inflation factor lambda (FASTA and GRAMMA); Sig, significance after Bonferroni correction (*, *P* < 0.05; **, *P* < 0.01; ***, *P* < 0.001). GeneID, closest SNP homology from BLAST. Tests were considered suggestive when *P* < 0.01 before Bonferroni correction.

**Table 5:** **Suggestive and significant QTL for trait *dead or alive *after challenge with *A. hydrophila *detected using PLINK (ASSOC) and GenAbel (FASTA and GRAMMA) analyses in 21** ***L. rohita *families**

**LG**	**Pos**	**SNP**	**Test**	**N**	**Effect**	**Stat**	***P*-value**	**Sig**	**GeneID**
1	37.3	55086_181	ASSOC	1022	0.102/0.146	9.07	0.00685		Small heat shock
1	37.3	61478_69	GRAMMA	979	-0.06 (0.03)	6.54	0.0095		
1	44.1	116899_232	GRAMMA	979	0.07 (0.03)	6.69	0.00875		
1	44.1	116899_232	ASSOC	1022	0.447/0.393	6.08	0.00625		
3	29	89585_200	ASSOC	1022	0.27/0.329	8.62	0.00548		Cardiac ankyrin repeat protein
3	29	89585_200	GRAMMA	979	-0.07 (0.02)	6.9	0.00772		Cardiac ankyrin repeat protein
4	0.9	32879_80	FASTA	979	0.08 (0.03)	7.86	0.00505		
4	0.9	32879_80	GRAMMA	979	0.08 (0.03)	8.44	0.00321		
5	23.8	83820_94	GRAMMA	979	-0.07 (0.03)	6.71	0.00863		Brain specific kinase 146
6	46.9	67578_280	FASTA	979	0.15 (0.05)	9.04	0.00264		Nicotinamide nucleotide transhydrogenase
6	46.9	67578_280	GRAMMA	979	0.15 (0.05)	9.71	0.00158	*	Nicotinamide nucleotide transhydrogenase
6	46.9	67578_280	ASSOC	1022	0.097/0.063	8.03	0.00397		Nicotinamide nucleotide transhydrogenase
6	46.9	87896_2052	FASTA	979	0.17 (0.06)	7.43	0.00643		Complement protein component c7-1
6	46.9	87896_2052	GRAMMA	979	0.17 (0.06)	7.97	0.00419		Complement protein component c7-1
7	53.7	87974_385	FASTA	979	-0.08 (0.03)	7.18	0.00738		sec14-like 1 (cerevisiae)
7	53.7	87974_385	GRAMMA	979	-0.08 (0.03)	7.71	0.00487		sec14-like 1 (cerevisiae)
7	53.7	87974_385	ASSOC	1022	0.246/0.3	7.4	0.00568		sec14-like 1 (cerevisiae)
9	32.1	56368_90	GRAMMA	979	-0.07 (0.02)	6.98	0.00739		leucine-rich ppr-motif containing
10	26.1	54734_19	FASTA	979	0.08 (0.03)	6.75	0.00937		
10	26.1	54734_19	GRAMMA	979	0.08 (0.03)	7.25	0.00633		
10	34.8	133884_276	GRAMMA	979	-0.08 (0.03)	7.02	0.0072		
13	26.9	65946_186	GRAMMA	979	-0.1 (0.04)	6.48	0.00985		Cytoskeleton associated protein 5
13	26.9	65946_186	ASSOC	1022	0.126/0.164	6.03	0.0083		Cytoskeleton associated protein 5
13	34.9	55609_284	FASTA	979	0.08 (0.03)	7	0.00816		Heavy subunit
13	34.9	55609_284	GRAMMA	979	0.08 (0.03)	7.51	0.00544		Heavy subunit
14	1.9	132996_241	FASTA	978	0.07 (0.02)	8.22	0.00415		
14	1.9	132996_241	GRAMMA	978	0.07 (0.02)	8.82	0.00259		
14	1.9	132996_241	ASSOC	1022	0.481/0.416	8.83	0.00448		
14	12.5	112228_90	FASTA	979	0.08 (0.03)	9.4	0.00217		
14	12.5	112228_90	GRAMMA	979	0.08 (0.03)	10.1	0.00127	*	
14	12.5	112228_90	ASSOC	1022	0.33/0.269	8.99	0.00334		
14	24.1	96692_167	FASTA	979	0.07 (0.02)	8.87	0.0029		Novel protein prickle-like family
14	24.1	96692_167	GRAMMA	979	0.07 (0.02)	9.52	0.00175	*	Novel protein prickle-like family
14	24.1	96692_167	ASSOC	1022	0.446/0.515	9.74	0.00588		Novel protein prickle-like family
14	47.1	4460_67	ASSOC	1022	0.372/0.46	8.29	0.0003	**	
15	28.9	60130_224	FASTA	978	-0.08 (0.03)	8.64	0.00328		
15	28.9	60130_224	GRAMMA	978	-0.08 (0.03)	9.28	0.00201		
15	28.9	60130_224	ASSOC	1022	0.32/0.377	7.21	0.005		
15	29.1	4834_117	FASTA	979	-0.07 (0.03)	6.92	0.00853		Novel protein
15	29.1	4834_117	GRAMMA	979	-0.07 (0.03)	7.43	0.00571		Novel protein
18	21.5	100422_182	ASSOC	1022	0.366/0.31	7.16	0.00972		
18	35.5	75070_130	FASTA	979	0.1 (0.03)	10.37	0.00128	*	
18	35.5	75070_130	GRAMMA	979	0.1 (0.03)	11.13	0.00072	*	
18	35.5	75070_130	ASSOC	1022	0.241/0.183	10.12	0.00095	*	
19	8.5	63493_143	GRAMMA	979	-0.06 (0.02)	6.54	0.00948		
19	23.8	111569_63	FASTA	979	0.11 (0.03)	10.88	0.00097	*	tbt-binding protein
19	23.8	111569_63	GRAMMA	979	0.11 (0.03)	11.68	0.00053	*	tbt-binding protein
19	23.8	111569_63	ASSOC	1022	0.181/0.126	11.86	0.00106	*	tbt-binding protein
20	1.4	115437_120	GRAMMA	979	-0.1 (0.04)	6.46	0.00994		myosin heavy chain
20	3	55229_133	ASSOC	1022	0.342/0.284	7.97	0.00459		denn madd domain containing 2d
20	3	55229_133	FASTA	979	0.07 (0.03)	7.75	0.00537		denn madd domain containing 2d
20	3	55229_133	GRAMMA	979	0.07 (0.03)	8.32	0.00344		denn madd domain containing 2d
20	3.3	134730_80	FASTA	970	-0.07 (0.02)	8.75	0.00309		
20	3.3	134730_80	GRAMMA	970	-0.07 (0.02)	9.39	0.00189	*	
20	3.3	134730_80	ASSOC	1022	0.332/0.398	9.43	0.00392		
20	9.4	110140_1196	FASTA	979	0.06 (0.02)	6.71	0.00959		glycerol-3-phosphate dehydrogenase
20	9.4	110140_1196	GRAMMA	979	0.06 (0.02)	7.21	0.00649		glycerol-3-phosphate dehydrogenase
20	9.4	110140_1196	ASSOC	1022	0.44/0.378	8	0.00638		glycerol-3-phosphate dehydrogenase
21	51.1	54579_132	ASSOC	1022	0.459/0.396	8.05	0.00374		
23	0	93296_256	ASSOC	1022	0.135/0.092	9.15	0.00121	*	loc795887 protein
23	0	93296_256	FASTA	978	0.11 (0.04)	6.69	0.00967		loc795887 protein
23	0	93296_256	GRAMMA	978	0.11 (0.04)	7.19	0.00656		loc795887 protein
24	49	110996_58	ASSOC	1022	0.204/0.26	9.14	0.00508		
24	49	110996_58	GRAMMA	979	-0.07 (0.03)	6.9	0.00773		
24	51.5	110996_644	ASSOC	1022	0.086/0.124	7.95	0.00843		
24	55.9	67606_298	ASSOC	1022	0.055/0.086	7.86	0.00404		serine long chain base subunit 1

LG, linkage group; Pos, location on LG in centimorgans; N, number of progeny and parents analysed; Effect, allele substitution effect of the minor allele with standard error in parenthesis (FASTA and GRAMMAS) or frequency of minor allele in case/control (ASSOC); Stat, chi-square with one degree of freedom for ASSOC, FASTA and GRAMMA analyses; P, point-wise empirical *P*-value (ASSOC) or permuted *P*-value with one degree of freedom corrected for inflation factor lambda (FASTA and GRAMMA); Sig, significance after Bonferroni correction (*, *P* <0.05; **, *P* <0.01; ***, *P* <0.001). GeneID, closest SNP homology from BLAST. Tests were considered suggestive when *P* <0.01 before Bonferroni correction.

Twelve SNPs mapping to six linkage groups (6, 14, 18, 19, 20 and 23, and covering possibly seven distinct regions in total, showed significant associations with the trait *dead or alive* (*P* <0.05 after Bonferroni correction, Figures 1B, D and F and 2B, D and F). One of these SNPs (4460_67 at 47.1 cM on LG14, with no known homology) was significant at *P* <0.01 after Bonferroni correction. A SNP mapping to this same position shares homology to chaperonin (HSP60) containing subunit 2 (132709_550, Additional file 1).

**Figure 4:**
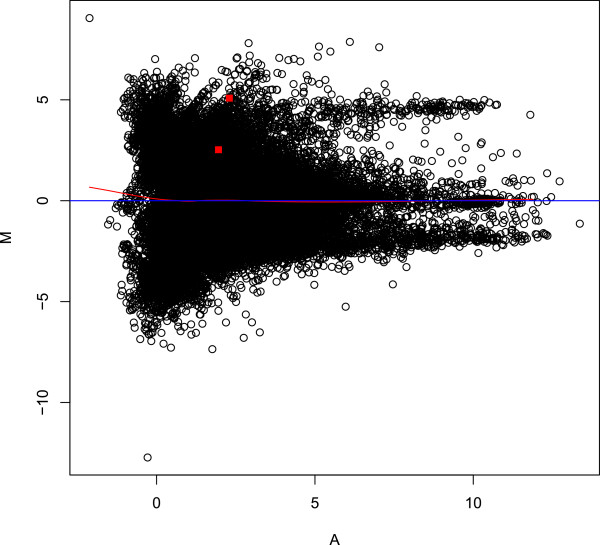
**Bland-Altman MA plots of quantile normalised log**_**2 **_**transformed coverage data.** Overall plot for 137,629 contigs (open circles) is overlayed with two highlighted contigs (shaded squares) showing homology to the mucin-5b precursor gene.

Of the SNPs with suggestive and significant associations with *hour of mortality* and *alive or dead* traits, several showed homology to genes of known immune function (Table 6). SNP 55086_181 at 37.3 cM on LG1 (*hours of survival* and *dead or alive* traits) showed homology to small heat shock protein, 87896_2052 at 46.9 cM on LG6 (*dead or alive*) to complement protein component c7-1, 31265_40 at 54.9 cM on LG8 (*hours of survival*) to CD22 antigen, 113696_50 at 43.9 cM on LG14 (*hours of survival*) to perforin 1, 110434_333 at 26.7 cM on LG15 (*hours of survival*) to t-cell antigen receptor alpha chain c region, 115737_104 at 23.8 cM on LG 16 (*hours of survival*) to mucin-5b precursor, 111569_63 at 23.8 cM on LG19 (*hours of survival* and *dead or alive*) to tributyltin (tbt)-binding protein and 554_399 at 23.2 cM on LG22 (*hours of survival*) to dipeptidyl-peptidase 7 (Tables 4, 5 and 6). Two contigs coding for mucin-5b precursor were found to be on average 3.8 times more highly expressed in resistant line than susceptible line fish (interquartile range 1.28, Figure 4), with contig_115737 (containing SNP 115737_104) around 5 times more differentially expressed in resistant line fish.

**Table 6:** SNPs with homology to genes of putative immune function mapping near to QTL regions

**QTL**		**Closely mapping SNPs of putative immune function**						
**LG**	**cM**	**cM**	**SNP**	**GeneID**	**Length**	**Hits**	**E-value**	**Similarity**
1	37.3 and 44.1	32.1	53470_163	Heat shock protein 105kd	615	10	9.33E-87	63.30%
		32.9	52852_1499	Complement c4	1991	10	0	62.60%
		37.3	55086_181	Small heat shock protein	857	10	6.64E-44	81.40%
2	43.9, 47.4 and 48.4	48	16321_60	Integrin alpha fg-gap repeat	127	3	5.33E-12	84.33%
3	20.3 and 29	27.4	134389_297	lymphocyte-specific protein tyrosine kinase	435	10	2.66E-64	89.90%
4	0, 0.9, 19.7 and 46.8*	-	-	-	-	-	-	-
5	9.6, 23.8, 38.3 and 43.4	9.6	98520_125	proteasome subunit beta type-6 precursor	168	10	9.08E-20	93.90%
		13	111876_59	major histocompatibility locus I antigen	250	10	9.21E-33	89.10%
		18	53025_556	c-type lectin receptor c	1141	10	7.40E-106	62.40%
6	46.9*	46.9	87896_2052	complement protein component c7-1	2667	10	0	71.50%
7	23.4** and 53.7	22.6	110314_603	e3 ubiquitin ligase	888	10	2.19E-138	66.90%
		54.4	134666_118	immunity related gtpase e4	203	10	2.16E-21	66.70%
8	23.3 and 54.9	54.9	31265_40	CD22 antigen	128	1	1.66E-05	71.00%
9	32.1	-	-	-	-	-	-	-
10	26.1, 29.6 and 34.8	27.9	117051_67	ubiquitination factor e4b isoform 2	116	10	9.21E-12	97.00%
12	31.1 and 37.8	-	-	-	-	-	-	-
13	19.8, 26.9 and 34.9	31	17842_95	mucin 2 protein	172	10	7.15E-17	80.70%
		31	53178_329	mucin 2 protein	2002	10	0	59.10%
		31	69593_98	mucin subtype tracheobronchial	307	10	2.79E-45	79.30%
14	1.9*, 12.5*, 24.1*, 43.9* and 47.1**	43.9*	113696_50	perforin 1	233	10	1.23E-24	67.10%
		47.1	132709_550	chaperonin (HSP60) containing subunit 2	922	10	6.24E-123	95.60%
15	1.1, 12.5*, 26.7, 28.9, 29.1 and 54.4*	26.7	110434_333	t-cell antigen receptor alpha chain c region	381	10	6.69E-31	74.00%
16	23.8 and 39.2	23.8	115737_104	mucin-5b precursor	347	3	9.86E-06	47.00%
18	8.4, 21.5, 35.5*, 48.9 and 49.9*	18.2	133571_269	MHC class II antigen beta chain	391	10	2.05E-32	76.50%
		36.7	52577_884	heat shock protein 70	2527	10	0	92.60%
19	23.8*	23.8*	111569_63	tributyltin (tbt)-binding protein	393	10	1.91E-46	50.50%
20	0.8, 1.4, 3, 3.3*, 7.9, 9.4, 11.1*, 20.3 and 21.6	-	-	-	-	-	-	-
21	47.1 and 51.1*	45.2	2465_218	ubiquitin-conjugating enzyme e2 c	279	10	1.75E-31	95.30%
22	6, 6.4, 22.3 and 23.2	22.9	83239_350	fish virus induced trim protein	447	10	3.79E-66	65.10%
23	0***, 0.9 and 27.4	0.7	55156_84	dermatin sulphate epimerase	378	10	4.94E-66	80.30%
		23.2	554_399	dipeptidyl-peptidase 7	502	10	1.19E-67	76.00%
24	49, 51.5 and 55.9*	-	-	-	-	-	-	-
25	46.1	-	-	-	-	-	-	-

GeneID, identity allocated by blast2go using consensus annotations for the top hits. Length, length of query contig sequence. Hits, number of sequences found to match query (maximum 10). E-value, minimum e-value (probability of alignment occurring by chance) recorded for a hit. Similarity, percent mean similarity recorded across hits. *, *P* <0.05; **, *P* <0.01; ***, *P* <0.001 after Bonferroni correction.

In addition to these noteworthy SNPs, some regions containing SNPs showing suggestive or significant associations with *hour of mortality* and/or *alive or dead* traits also contained candidate genes of interest with respect to disease resistance (Table 6). SNPs with homology to complement c4 (52852_1499) and heat shock protein 105kd (53470_163) map approximately 5 cM from small heat shock protein 55086_181 on LG1 (Additional file 1) which has suggestive associations with both traits (Tables 4, 5 and 6). SNP 16321_60 with homology to the integrin alpha fg-gap repeat maps to 48 cM on LG2, within 1 cM of three SNPs with suggestive associations on *hours of survival* (Additional file 1, Table 4). SNP 134389_297 with homology to lymphocyte-specific protein tyrosine kinase maps approximately 2 cM from SNP 89585_200 (suggestive association with *dead or alive*) on LG3 (Additional file 1, Tables 5 and 6). SNP 98520_125 with homology to proteasome subunit beta type-6 precursor maps to the same position, 9.6 cM along LG5, as SNP 4797_109 (suggestive association with *hours of survival*) (Additional file 1, Tables 4 and 6). SNPs 111876_59 and 53025_556 with homology to the major histocompatibility locus I antigen (MHC I) and the c-type lectin receptor c map to 13 cM and 18 cM on LG5 respectively 3.4 and 5.8 cM from SNPs 4797_109 (suggestive association to *hours of survival* for the GRAMMA and FASTA tests, Additional file 1, Tables 4 and 6) and 83820_94 (suggestive association with both *hours survival* and *dead or alive*) respectively (Additional file 1, Tables 4, 5 and 6). A SNP with homology to e3 ubiquitin ligase (110314_603) occurs at the same location as SNP 62374_157 (significant association with *hours of survival*, FASTA *P* <0.05 and GRAMMA *P* <0.01), while another SNP with homology to immunity related gtpase e4 (134666_118) maps to the same position as SNP 87974_385 (sec14-like 1, suggestive associations with *hours of survival* and *dead or alive*) on LG7 (Additional file 1, Tables 4, 5 and 6). SNP 117051_67 with homology to ubiquitination factor e4b isoform 2 maps between two SNPs with suggestive associations, 1.7 cM distant from SNP 82862_249 (*hours of survival*) and 1.8 cm distant from 54734_19 (*dead or alive*) on LG10 (Additional file 1, Tables 4 and 6). SNPs 17842_95, 53178_329 and 69593_98 all share homology with mucin 2 protein and map 3.9 cM from SNP 55609_284 (suggestive association with *dead or alive*) on LG13 (Additional file 1, Tables 5 and 6). SNP 110434_333 with homology to the alpha chain c region of the T cell antigen receptor (suggestive associations with *hours of survival*) maps 2.2 cM from SNPs 54100_91 (suggestive associations with *hours of survival*) and 60130_224 (suggestive association with *hours of survival* and *dead or alive*) on LG15 (Additional file 1, Tables 4, 5 and 6). SNP 133571_269 with homology to MHC class II antigen beta chain maps 3.2 cM from 100422_182 (suggestive association with *dead or alive*) and SNP 52577_884 with homology to heat shock protein 70 maps 1.2 cM from SNP 75070_130 (significant association with *dead or alive*, *P* <0.05 after Bonferroni correction for ASSOC, FASTA and GRAMMA) on LG18 (Additional file 1, Tables 5 and 6). SNP 2465_218 with homology to ubiquitin-conjugating enzyme e2 c maps 2.1 cM from SNP 111636_59 (suggestive association with *hours of survival*) on LG21 (Additional file 1, Tables 4 and 6). SNP 83239_350 with homology to fish virus induced trim protein maps between two SNPs with suggestive associations, 0.3 cM from 554_399 and 0.6 cM distant from SNP 58881_141 (*hour of survival*) on LG22 (Additional file 1, Tables 4 and 6).

### Temporal gene expression changes with *A. hydrophila* infection

**Figure 5:**
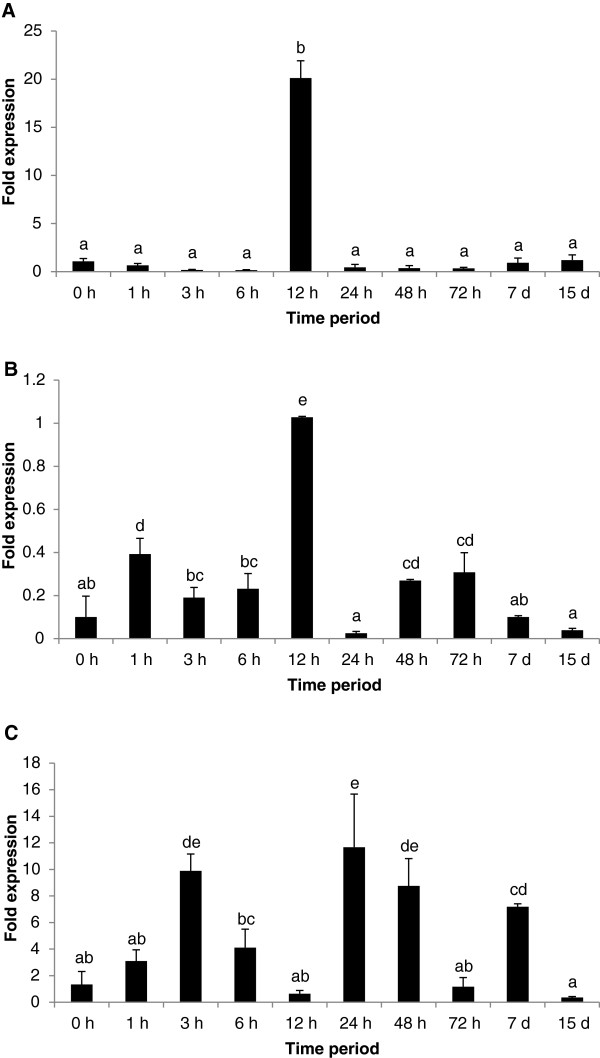
**Temporal expression analysis of the perforin gene in spleen (A), liver (B) and gill (C) tissues of *L. rohita *hours (h) and days (d) after infection with *A. hydrophila*.** Fold expression was calculated as 2^-∆∆Cq^. The control group (0 hour post-challenge) was used for calibration. Bars bearing different superscript are significantly different (*P* < 0.05).

Significant up-expression of the perforin gene was observed over the time course post-infection with *A. hydrophila*, particularly in rohu spleen and gill tissues (Figure 5). Perforin was highly up-expressed (20-fold) at 12 h post-infection. The expression level in spleen did not significantly differ from pre-infection levels over the rest of the time periods sampled. Up-expression in the liver began 1 hour post-infection infection (0.4 fold), was highest at 12 h post-challenge (1 fold), dropped to pre-infection levels at 24 h and again slight up-regulation was noticed from 48 – 72 h post-infection (0.27-0.3 fold). Expression levels in gill tissue fluctuated over the time course, with up-expression at 3 h (9 fold), reduced levels of expression at 6 h and 12 h, increasing to the highest level at 24 h (11 fold), remaining high at 48 h (8 fold), decreasing to pre-infection levels at 72 h and increasing again at 7 d post-infection (7 fold).

## Discussion

This study created an extensive new SNP linkage map resource for *L. rohita* that was used to scan the genome for polymorphisms associated with *A. hydrophila* resistance. The SNPs occur in transcribed genes and were detected by looking for variation within and between populations of *L. rohita* that were differentially selected for either resistance or susceptibility to *A. hydrophila*[9]. This strategy was taken to maximise the possibility that some of the SNPs detected were the actual causative variants, or, that they would map closely to the actual causative gene variants affecting resistance to *A. hydrophila*.

### Linkage groups and genome coverage

A dense genetic linkage map for *L. rohita* was created containing 3193 SNPs mapping to 25 linkage groups (Additional file 1) corresponding to the haploid number of chromosomes in *L. rohita*[12]. Other linkage mapping studies for carps have estimated map lengths of widely differing sizes. For example, common carp map sizes range from 1852 cM to 5506 cM (between 161 and 719 and markers were used in these studies generating between 42 and 64 linkage groups [13-16]). Many of these size estimates are likely to be inaccurate due to poor coverage. Genome lengths for the male and female maps in this study were 1407 and 1416 cM respectively. The haploid genome of rohu has been estimated to consist of approximately 1950 million base pairs (based on the Feulgen microdensitometry method) [17]. Given the genome length estimate from our study we would therefore expect approximately 1.4 million bases per cM distance in the common carp genome. Pairwise recombination rates between informative linked markers were not significantly different for male compared to female meiosis.

More than 90% of BLAST search homology for the SNP annotation was with genes in the zebra fish (*Danio rerio*) genome [9]. The correspondence between the organisation of these two genomes was determined by comparing the linkage map positions of annotated *L. rohita* SNPs to the chromosomal position of the same genes in *D. rerio*. Some chromosomal rearrangement since these species diverged from a common ancestor some 10–250 million years ago [18] was observed (Additional file 3). A few scattered genes mapped to different *D. rerio* chromosomes than neighbouring genes on the *L. rohita* linkage map. This may be due to actual chromosome rearrangements (eg. caused by transposable elements) or due to errors of identification caused by BLAST similarity with closely related gene sequences on other chromosomes. Most of these differences occur towards the end of the *L. rohita* linkage groups which is also where rearrangements of large blocks of genes within linkage groups/chromosomes were detected (eg. towards the end of linkage group 18 in *L. rohita*). The gene sequences themselves shared high similarity (average similarity 87 ± 0.3% SE).

### Candidate genes mapping to QTL regions

A number of SNPs in contigs with homology to genes of known immune function were found to either be the SNPs, or map closely (within 5 cM distance) to SNPs, with suggestive (*P* <0.01 before Bonferroni correction) or significant (*P* <0.05 after Bonferroni correction) associations with *hours of survival* and/or *dead or alive* traits after challenge with *A. hydrophila* (Tables 4, 5 and 6).

The ubiquitin-protein ligases or E3 enzymes (e3 ubiquitin ligase 1110314_603 on LG7, ubiquitination factor e4b isoform 2 117051_67 on LG10 and ubiquitin-conjugating enzyme e2 c 2465_218 on LG21, Table 6) are a diverse group of enzymes that, as part of an enzyme cascade, attach ubiquitin to a lysine on the target protein resulting in poly- or mono-ubiquination which targets specific protein substrates for degradation by the proteasome (proteolysis) [19]. More than 500 distinct E3 enzymes have been found in mammals. Silencing of ubiquitin ligase associated proteins has been shown to affect disease resistance in plants [20]. A SNP coding for e3 ubiquitin ligase occurs at the same map position around 23.4 cM on LG7 as SNP 62374_157 which is significantly associated with *hours of survival* (FASTA *P* <0.05 and GRAMMA *P* <0.01 after Bonferroni correction Additional file 1, Table 4).

The proteasome is a large complex which catalyses the degradation of ubiquitinated proteins, a process requiring ATP to unfold and translocate the substrate into the core of the proteasome for proteolysis [21]. The architecture of the proteasome ensures that only those molecules which are targeted for degradation are affected and the proteolytic enzymes at the core of the proteasome cleave peptide bonds with broad specificity. With degradation of intracellular proteins by the proteasome, some of the by-products are transported to the endoplasmic reticulum where they bind to major histocompatibility class I molecules and result in antibody production [22]. Variation in the proteasome subunit genes (eg. variation in the proteasome subunit beta type-6 precursor) which affects the structure and function of the proteasome could therefore have downstream effects on cellular immunity.

Lymphocyte-specific protein tyrosine kinase (SNP 134389_297 on LG 3, Table 6) is highly expressed in the thymus, initiates tyrosine phosphorylation cascade in T-cells and plays a crucial role in T-cell maturation, signalling and hence immunity [23-25]. The basic mechanisms that regulate expression of this gene have been shown to be highly conserved between teleost fish and mammals [26].

The major histocompatibility class I antigen (MHC I, 111876_59 on LG5, Table 6) alerts the immune system to the presence of foreign material inside a cell. MHC I presenting proteins (HLS’s) occur on the cell surface. The MHC II interacting molecule CD4 communicates with T-cell receptors, and it is MHC II (133571_269 on LG 18, Table 6) that is known to mainly fight bacterial pathogens [27], although MHC I has evolved MHC II type functionality in some fish species such as Atlantic cod *Gadus morhua*[28]. Three MHC class I alleles have been found to be associated with improved resistance and four MHC class II alleles were found to be associated with increased susceptibility of Atlantic salmon to *Aeromonas salmonicida* infection [29]. Fixed allele frequency differences were detected for several MHC I SNPs, including SNP 111876_59 which mapped 3.4 cM from the QTL detected on LG5, between samples from lines of rohu that were selected for resistance or susceptibility to *A. hydrophila*[9]. More than 5-fold up- or down-regulation of MHC I transcripts was also detected in the resistant line fish using mRNA-seq and differential expression was confirmed for one transcript (contig 88601) in the skin, gill and intestine using RT qPCR [9].

The highly variable alpha chain of the T cell receptor (110434_333 on LG15, Table 6) occurs on the surface of T lymphocytes, and along with the beta chain, recognises antigens bound to MHC molecules. Two c alpha chain molecules have been detected in common carp (possibly as a result of tetraploidisation) [30]. *A. hydrophila* has been found to significantly increase the expression of beta chain T cell antigen receptors in Nile tilapia peripheral blood leukocytes grown in culture [31]. Activation of invariant natural killer T cells, with an invariant T-cell antigen receptor alpha chain, have been proposed as attractive targets for developing new vaccines for infectious diseases because of their ability to recognise glycolipid antigens from pathogenic bacteria including *Streptococcus pneumonia*[32].

Heat shock 70 kDa (HSP70 or mortalin, 52577_884 on LG18, Table 6) binds to antigen presenting cells via toll-like receptors and leads to the secretion of pro-inflammatory cytokines and broad immunostimulation [33]. HSP70 acts as an intracellular chaperone, which stabilises proteins, giving them a possible role in general stress tolerance. This protein plays a role in cell proliferation, stress response and maintenance of the mitochondria. Seven contigs with homology to HSP70 were up-regulated more than 3-fold (median 4.89) in resistant compared to susceptible line rohu [9]. Heat shock protein 105/110 (53470_163 on LG1, Table 6) is a member of the HSP70 family of molecular chaperones which functionally relates to heat shock cognate protein 70 (HSC70) and HSP90, and is known to prevent the aggregation of denatured proteins in cells under severe stress [34]. HSP60 (132709_550 on LG14, Table 6), like HSP70, is believed to play an important role in the control of the immune response [35].

A number of genes with putative functions affecting mucous secretions (mucin-5b precursor 115737_104 on LG 16, mucin 2 17842_95, mucin 2 53178_329 and mucin subtype tracheobronchial 69593_98 on LG13, Table 6) were associated with QTL of significant or suggestive association with the two traits. Two rohu contigs with homology to the mucin-5b precursor gene [detected by 9] were on average 3.8 times more highly expressed in resistant line than susceptible line fish (interquartile range 1.28, Figure 4). Five contigs with homology to mucin 2 [detected by 9] were on average 2.29 more highly expressed in resistant line than susceptible line rohu (interquartile range 0.362). High molecular weight glycoprotein polymers called mucins are found on the outer body surfaces and intestine of fish. These glycoproteins form a highly viscous gel that protects the epithelium from microbial and other disturbances. Common carp increase the amount and total glycosylation of high molecular weight glycoproteins in the skin in response to increased bacterial loads [36,37]. Twenty-six contigs with homology to zona pellucida glycoprotein have been found to show two- to seven-fold higher expression (median 4.13, interquartile range 2.45) in resistant compared to susceptible line rohu [9]. Choriogenin is another high molecular weight glycoprotein and was found to be 3.5 times more highly expressed in resistant compared to susceptible line rohu [9]. Higher expression of these glycoprotein genes could result in the secretion of greater quantities of glycoproteins, including mucin, on the skin and gut surface leading to greater protection and readiness against bacterial disease.

Serum lectins (c-type lectin receptor c 53025_556 on LG5, Table 6) are found in the mucus and have been shown to agglutinate with and alter the viability and pathogenicity of Gram-negative bacteria including *A. hydrophila*[38-41]. Seven-fold higher expression of the serum lectin isoform 1 precursor gene was detected in resistant line than susceptible line rohu, and alternative isoforms of galactoside-binding soluble lectin 9 in resistant and susceptible line rohu were detected by [9]. Along with a higher production of mucin, higher production of lectins found in the mucus of the skin and gut could lead to a greater preparedness to combat and resist infection by bacterial pathogens. Cluster of differentiation 22 (CD22 antigen 31265_40 on LG8, Tables 4 and 5) is a lectin that is found on the surface of mature B cells and prevents over activation of the immune system [42]. CD22 is a negative regulator of antigen receptor signaling in B cells. In mice CD22 is down-regulated on wild-type B-1 cells in response to LPS [43].

Tributyltin binding protein (111569_63 on LG 19, *P* <0.05 after Bonferroni correction for FASTA and GRAMMA tests for *hours of survival* and FASTA, GRAMMA and ASSOC tests for *dead or alive*, Tables 4 and 5) is a glycoprotein (possible lipocalin) that is believed to be involved in the transportation, detoxification and excretion of xenobiotic compounds such as tributyltin in the blood of Japanese flounder [44]. Tributyltin-binding protein is excreted from the body of Japanese flounder via the skin mucus. Tributyltin-binding protein is up-expressed more than four fold in the spleen of turbot 3 days after challenge with *Aeromonas salmonicida*[45].

Complement protein component c7 (87896_2052 on LG6, Table 5) plays an important role in the membrane attack system of the innate and adaptive immune response by serving as a membrane anchor, facilitating the formation of pores in the plasma membrane of target cells [46]. In a condition known as complement component 7 deficiency, human patients are more susceptible to recurrent infections, particularly to bacterial diseases such as caused by meningococcal infection [47].

Other genes with putative immune function with SNPs of interest included pore forming protein (perforin 1 113696_50 on LG 14, *P* <0.05 after Bonferroni correction for the GRAMMA test of *hours of survival*, Table 4) which is a key molecule involved in T-cell and natural killer-cell-mediated cytolysis, inserting itself into the target membrane forming a pore which allows cytolytic proteins to enter the cell and trigger it to self-destruct [48], dipeptidylpeptidase 7 (554_399 on LG22, Table 4) which suppresses apoptosis of resting lymphocytes [49] and immunity related gtpase e4 (134666_118 on LG 7, Table 6) which is one of a family of proteins that are activated as part of an early immune response (induced by interferon) and that localises to and disrupts the phagocytic vacuole during infection. Large temporal changes in perforin gene expression post-infection were detected by quantitative real-time PCR in spleen (up to + 20 fold at 12 h post-infection, Figure 5a) and gill tissue (9, 11, 8 and 7 fold at 3 h, 24 h, 48 h and 7 days post-infection, respectively, Figure 5c). Along with linkage and genome-wide association evidence for a QTL mapping to the perforin gene region on LG14 (Tables 3, 4 and 5), these patterns suggest that differential expression of the perforin gene itself could play an important role in the immune defense of *L. rohita* against *A. hydrophila* infection, and that polymorphisms affecting the expression of this gene during the time course of infection could influence disease resistance.

### Comparison of traits and tests

In all cases except one, the position of QTL mapped using half-sib regression analysis co-located within 10 cM of a nominally significant SNP in the GWAS analysis for the two traits. In two cases on LG15 at 29 cM and LG23 at 0 cM, the QTL peak from the linkage analysis (Table 3) co-located to the same position as significant SNPs in the GWAS analysis for the *hours of survival* trait (Table 4). There was good correspondence between the results for the ASSOC, FASTA and QFAM GWAS test results (most suggestive/significant results were detected by >1 GWAS test). As the challenge tests were performed for the different families over different total time frames it was not valid to compare *hours of survival* between families using the “total” option in qfam. The concordance between the findings for the *hours of survival* and *dead or alive* trait analyses was fairly high. Overall, many of the same regions (on linkage groups 1, 4, 5, 7, 10, 14, 15, 19, 20, 21, 23 and 24) contained SNPs with suggestive or significant associations for both the *hours of survival* and *dead or alive* traits (Tables 4 and 5), heritability estimates for both traits were similar (0.05 and 0.07 respectively) and the genetic correlation between the two traits was high and positive (0.79), indicating that the same underlying genetic mechanisms may be affecting these traits.

As the heritability of *hours of survival* and *dead or alive* post-challenge with *A. hydrophila* is low (similar levels of heritability for *A. hydrophila* resistance have been found for common carp, 0.04) [50] this is likely to be a polygenic trait influenced by the small additive effects of many genes and by the environment. Polymorphisms affecting the regulation of expression or amino acid structure of the proteins expressed by the immune genes highlighted in this study could be the type of causative mutations contributing to overall *A. hydrophila* resistance in *L. rohita*. It is not possible to identify the causative mutations from the results of this current study, but the genes and linkage group regions highlighted here provide clues that will direct the focus of future investigations, and provide potentially useful loci for marker assisted selection to improve *A. hydrophila* resistance in this and related species.

## Conclusions

In summary, an important resource has been developed and used to screen the *L. rohita* genome for quantitative trait loci associated with disease resistance. mRNA-seq was used by a previous study to identify SNPs in transcribed genes using samples from susceptible and resistant line rohu [9]. In the current study 3194 of these SNPs were mapped to 25 linkage groups (average interval distance of 1.3 cM) and used to detect quantitative trait loci associated with resistance to *A. hydrophila*. Associations with resistance to *A. hydrophila* of significance after Bonferroni correction mapped to linkage groups 4, 6, 7, 14, 15, 18, 19, 20, 21, 23 and 24. Genes of immune function mapping closely to these QTL include heat shock protein 70, heat shock protein 60, heat shock protein 105, “small heat shock protein”, mucin 5b precursor, mucin 2, lectin receptor, CD22, tributyltin-binding protein, major histocompatibility loci I, major histocompatibility II, complement protein component c7-1, perforin, ubiquitin ligase, ubiquitination factor e4b isoform 2, ubiquitin-conjugation enzyme e2 c, proteasome subunit, T-cell antigen receptor and lymphocyte specific protein tyrosine kinase. The SNPs detected in association with disease resistance traits could be useful markers for improving the disease resistance of farmed rohu populations with selective breeding. They may also prove to be useful markers for Aeromoniasis resistance in closely related species such as common carp. Further work should be undertaken to confirm the associations detected in rohu carp and to determine whether the results are transferrable to other carp species. Genes of putative immune function mapping closely to these QTL provide leads for future work aiming to improve our understanding of the causative genetic variants affecting disease resistance. Knock-out gene studies on rohu, or on closely related model species such as zebra fish, could be used to determine whether the regulation of expression of these genes affects disease resistance, or whether other functional differences are involved. Elucidating the precise immune function of these genes will be a challenge, but such knowledge could hold large benefits for the world’s aquaculture production, which relies so heavily on carp species affected by this disease.

## Methods

### *A. hydrophila* challenge experiment

The rohu fingerlings used for the study were generated under a selective breeding program initiated at the Central Institute of Freshwater Aquaculture (CIFA) India in 1992 under an Indo-Norwegian collaboration between CIFA and Institute of Aquaculture Research (AKVAFORSK), Norway. The original founders of the breeding program were sourced before 1992 as fry or fingerlings from five rivers in India (the Ganga, the Gomati, the Yamuna, the Brahmaputra and the Sutlej) [51,52]. All fingerlings for this study were derived from the 2008 year class of the breeding program which had been selected over seven generations for increased growth rate. Twenty-one full-sibling families were represented. Different sire and dam individuals were pair mated to produce each family. However, four of the parents were themselves derived from two full-sib dam families. The parents were from the sixth generation of the selective breeding program (2005 & 2006 year classes). The families were challenge tested during April–May, 2009. Five hundred juvenile rohu fingerlings from each family were collected from the nursery ponds, transferred to cement tanks (10 × 5 m^2^) and left for two weeks acclimatization. Fish were fed a standard commercial pellet diet (3% of body weight in two doses daily). One tenth of the water in the tank was exchanged daily for the removal of faecal material and unused feed. An overnight culture of *A. hydrophila* was grown in tryptone soya broth at 30°C for 20 h. Fish were challenged with the same pathogenic strain of *A. hydrophila* intraperitoneally at an LD50 dose of 2 × 10^6^ cfu/20 g fish. The LD50 bacterial dose required was calculated prior to the experiments using a separate representative sample of fingerlings collected from all families. Hourly observations of mortality for individuals in each family were recorded for up to 10 days. Liver and/or muscle tissue from 100 early death and 100 late death or surviving animals were collected aseptically from each family and kept in ethanol at -20°C for further DNA extraction. All challenge trials were approved by an Institutional Ethics Committee and performed in accordance with the Indian Government Prevention of Cruelty to Animals Act 1960.

### DNA extraction

Out of the 100 early death and late death/surviving fish in each family, sixty muscle tissue samples from each category were randomly chosen and processed for DNA extraction. Before extraction of DNA, tissue samples (50–100 mg) were rinsed vigorously with 1 ml of PBS and cut into fine pieces with sterile scissors. The minced tissues were processed further for extraction of DNA following a high salt method (http://www.genomics.liv.ac.uk/animal/RESEARCH/ISOLATIO.PDF). The extracted genomic DNA was dissolved in TE buffer (10 mM Tris Cl, 1 mM EDTA) and stored at -20°C. The Phenol Chloroform method described by Sambrook et al. [53] with slight modifications was used to further purify the DNA. The quality of extracted DNA was checked on a 2% agarose gel in 1X TBE buffer after electrophoresing at 50 V for an hour. The concentration and purity of DNA was checked using OD values at 260 and 280 nm. Quantification was achieved using the OD value at 260 nm measured by a Nanodrop 2000C (Thermo Scientific).

### SNP array

A custom design Illumina iSelect SNP-array was manufactured by Illumina (Illumina, San Diego) containing 6000 candidate SNP loci identified in the *de novo* assembled transcriptome [9]. SNPs were identified by two numbers separated by an underscore, where the first number was the contig identification number, and the second number was the SNP position in base numbers along the contig length. SNPs chosen for the array had the following characteristics, (i) putative minor allele frequency greater than 0.33 based on sequencing data, (ii) contig lengths greater than 200, (iii) only one SNP per contig, (iv) compatible for the Infinium II Assay (i.e. A/G, A/C, T/G or T/C), and (v) Assay Design Tool (ADT) scores greater than 0.85. Samples (n = 1152) were genotyped following standard protocols for the iSelect SNP-array (Illumina, San Diego). Genotypes were called by first performing automatic clustering using Genome Studio Genotyping Module (V1.9.4), and then by excluding low call rate (low quality) samples, introducing pedigree information and performing manual SNP cluster correction to improve calling where necessary. Manual checking was performed to facilitate the subjective classification of individual marker assays into categories including “failed”, “monomorphic”, and “SNP” to ensure that only high quantity SNP calls were included in subsequent analysis.

### Genomic pedigree checks on the family material used for mapping

SNP genotypes were grouped based on estimated pairwise relatedness values using the COANCESTRY software package [54] to confirm the parentage assignment of offspring determined at tagging. Following pedigree relationship clustering, the corrected pedigree data (genomic pedigree) was checked for Mendelian inheritance errors using a script written by one of the authors in R and SNPs or animals showing consistent errors were removed from further analysis.

### Linkage map

A linkage map of the SNP markers was constructed using the full-sib families with available parental genotypes (seven families containing between 114 and 28 offspring) and the software TMAP http://users.math.yale.edu/~dc597/tmap/[55]. Initially, the program *phasing* was used to define the marker phases in each family in the pedigree. Subsequently, *pedmerge* was used to merge these multiple phase-known pedigrees into a single data file. *Grouping* was used to identify groups of linked markers, with the LOD threshold varied until the number of groups reflected the expected number of chromosomes. Finally, *tmap* was used to order the markers within each linkage group. Both sex-specific and sex-averaged linkage maps were generated. Graphics of the linkage groups were generated with MAPCHART software [56].

Goodness of fit G tests were used to test for segregation distortion (proportions differing from segregation ratios expected with Mendelian inheritance) within families for each SNP using a chisq.test function in R. A Bonferonni correction (based on the number of linkage groups examined, which was 25 for *L. rohita*) was applied to limit experiment-wide error rates associated with multiple testing [57].

### Comparison of genome organisation to zebra fish (*Danio rerio*)

To annotate the *L. rohita* linkage map with *D. rerio* chromosome locations, protein coding sequences from *Danio rerio* were downloaded as Fasta sequences from Ensembl (26 July 2013) together with their corresponding chromosome location. BLASTx (version 0.0.1 in Galaxy) was then used to find the single best match between the *Danio rerio* protein coding sequence and the sequences from the *L. rohita* contigs which contained mapped SNPs (e-value <1.00E-10).

### Genetic parameters, the significance of fixed effects and correlation of traits

Two alternative phenotypic traits were considered for analysis, (i) *hours of survival*, which is a continuous trait, and (ii) *early death* vs. *late death or survival* (hereafter referred to as *dead or alive*), which is a binomial trait where animals that were among the first 100 to die from each full-sibling family were classified as *dead* and animals among the last 100 to die or survive the full duration of the challenge were classified as *alive*.

An animal model was applied to estimate genetic parameters and the significance of fixed effects (without accounting for SNP genotype). A Markov chain Monte Carlo (MCMC) method using a multi-trait generalised linear mixed effect model (glmm) in a Bayesian estimation framework, with animal breeding value fitted as a random effect, was used for the analysis R Package, MCMCglmm, [58], http://www.cran.r-project.org. Our main interest was whether tank and/or family should be included as fixed effects in the QTL analysis. All offspring were juveniles when challenged and sex could therefore not be determined. Animal and ID were fitted as random terms. ID was the same as the animal factor, but was used by MCMCglmm to dissociate individual records from the pedigree and give an indication of between individual variance [59]. Therefore the model fitted was as follows,

y=mu+tank+family+animal+ID

where *y* was *hours of survival* or *dead or alive*, *tank* and *family* were fixed effects, *animal* and *ID* were random animal effects and *mu* represented unknown random residual effects.

All models were run using 300,000 iterations as burn-in, 1 million iterations for sampling and a thinning interval of 500. A “plausible” prior assuming weak genetic control (additive genetic variance, permanent environmental variance and residual variance accounting for 0.2, 0.1 and 0.7) was used with the smallest possible degree of belief parameter (n = 1).

Estimates of additive genetic variance and residual variance were calculated from the modes of the posterior distribution and a Bayesian equivalent of 95% confidence intervals was obtained by calculating the values of the estimates that bound 95% of the posterior distributions. Narrow sense heritability (*h*^*2*^), or the proportion of total phenotypic variance that is additive genetic in origin, was estimated under the model as *V*_*A*_ / (*V*_*A*_ + *V*_*E*_ + *V*_*e*_) where *V*_*A*_,*V*_*E*_ and *V*_*e*_ were variance attributed to additive genetic, permanent environmental effects unconstrained by pedigree and residual error effects respectively. In a similar fashion, the additive genetic correlation between the traits *hours of survival* (*x*) and *dead or alive* (*y*) (or proportion of variance the two traits share due to additive genetic causes, *r*_*Axy*_) was estimated as $\mathrm{CO}V_{\mathrm{Axy}}/\sqrt{\left[V_{\mathrm{Ax}}^{2}V_{\mathrm{Ay}}^{2}\right]}$ where COV_Axy_ was the genetic covariance for the traits and V_Ax_ and V_Ay_ were the additive genetic variances attributed to *hours of survival* and *dead or alive* traits respectively. Heritability and additive genetic correlation between the traits was considered significant if the 95% credible interval of the posterior distribution did not span zero.

### QTL linkage analysis

Both the binary *dead or alive* and continuous *hours of survival* trait were used for linkage analysis. QTL detection was carried out using a linear regression interval mapping approach in GridQTL [60]. The binary trait dead/alive was analysed together with the number of survival days in the challenge. It has been shown that a binary trait can be analysed using QTL mapping methods intended for quantitative traits, as long as the trait is a threshold trait with an underlying normal distribution [61,62]. Linkage analysis was performed separately for sires and dams of the full-sib families used in the study. *P*-values were calculated for all trait-by-chromosome combinations with the significance of the peak F-statistic (putative QTL) estimated after 10,000 chromosome-wide permutation tests. A QTL was found to be genome-wide significant if the chromosome-wide significance level was less than than 0.002 (0.05/25), a Bonferroni correction based on the number of chromosomes in rohu.

### Genome-wide association study

A genome-wide association study (GWAS) was performed using GenABEL (http://www.genabel.org) and Plink [http://pngu.mgh.harvard.edu/purcell/plink/ 59]. First we determined which markers and individuals should be excluded from the GWA analysis using the check.marker function in GenABEL. This function was used to exclude individuals or markers with call rate <95%, markers with minor allele frequency <0.24%, individuals with high autosomal heterozygosity (FDR <1%) and individuals with identity by state ≥0.95. Genomic kingship was computed between all pairs of individuals. We performed a pedigree based association analysis where the pedigree is a confounder (where the heritable trait is more similar between close relatives and therefore some degree of association is expected between any genetic marker and any heritable trait). The effect of the confounding pedigree is expected to inflate the resulting null distribution of the chi square test statistic by a constant, lambda. Lambda is a function of the traits heritability and pedigree structure (expressed as a kinship matrix). Two fast tests for genome wide association were applied, Family-based Score Test for Association (FASTA, [63]) and Genome-wide Rapid Analysis using Mixed Models And Score test (GRAMMAS, [64]) using the R package GenABEL (http://www.cran.r-project.org). A mixed polygenic model of inheritance was applied in order to study association in our genetically homogeneous families where the *hours of survival* or *dead or alive* traits (*y*) were modelled as:

y=μ+f+G+e

where μ is the intercept, *G* describes the polygenetic effect (contribution from multiple independently segregating genes all having a small additive effect on the trait), *f* describes fixed effects (where *f* was either *tank* + *family*, or where no fixed effects were included in the model) and *e* describes the random residual effects. The joint distribution of residuals in the pedigree was modelled using a multivariate normal distribution with variance-covariance matrix proportional to the identity matrix. A genomic kingship matrix, generated by calculating the average identity-by-state between individuals in the pedigree (ibs in GenABEL), was used as the relationship matrix for FASTA and GRAMMAS. Both FASTA and GRAMMAS exploit maximum likelihood estimates of the intercept from the polygenic model. Two hundred permutations were used to estimate genome wide significance for the FASTA and GRAMMAS test (downward bias in the estimate of SNP effects are expected).

The QFAM and ASSOC analysis modules in PLINK http://pngu.mgh.harvard.edu/purcell/plink/[65] were used to perform a linear regression of phenotype on genotype for the traits *hours of survival* and *dead or alive* respectively. In the case of QFAM, the module used a permutation procedure to correct for family structure. Association testing was performed within families. Ten thousand permutations per SNP (flipping allele transmission from parent to offspring with 50:50 probability) were applied producing a point-wise estimate of each individual SNPs empirical significance.

GWAS associations with significance at *P* < 0.001, *P* <0.01 and *P* <0.05 levels after Bonferroni correction based on the number of linkage groups (which was 25 for *L. rohita*) were noted for all tests.

### Differential expression in naïve susceptible and resistant line rohu

Differential transcript expression in selected *A. hydrophila* resistant versus susceptible rohu individuals was checked for some SNP containing contigs using sequence coverage data and methods collected and described in [9]. In brief, selection of the resistant and susceptible line fish by the previous study was made using intra-peritoneal challenge testing of 87 full sibling families (30 siblings per family) with a virulent strain of *A. hydrophila*. Unchallenged individuals from the 15 highest and 10 lowest ranking families were selected to create the first generation of the resistant and susceptible lines respectively. RNA pools were prepared from liver, intestine, muscle, kidney, spleen and brain tissue samples from 10 resistant and 10 susceptible line fish for comparison. Quantile-based rank normalisation was used to correct for differences between sequencer flow cells or RNA pools [66]. Data were visually represented as Bland-Altman *MA* scale plots where *M* = log_2_*R*-log_2_*S* and *A* = 0.5*(log_2_*R* + log_2_*S*), *R* and *S* being average coverage depth for each contig in the resistant and susceptible line pools respectively.

### Temporal gene expression changes with *A. hydrophila* challenge

Rohu juveniles from the 2008 year class were collected and kept in 700 L ferro-cement tanks for acclimatization prior to the experiment. A virulent strain of *A. hydrophila* (Ah 15) was isolated from a field outbreak that occurred on a farm at Puri, Odisha, India (Mohanty et al. 2008). Forty eight rohu juveniles (weighing 80–100 g) were challenged intra-peritoneally with live *A. hydrophila* at a dose of 1.5 x 10^6^ cfu/g of body weight. Tissue samples from liver, gill and spleen were collected from infected fish at different time periods viz., 1, 3, 6, 12, 24, 48, 72 hours, 7 and 15 days post-infection, along with three non-infected controls, in triplicate after euthanasia with a heavy dose of MS222. The tissue samples were kept in RNA*later* (Sigma, USA) and stored at -20°C until RNA extraction. Total RNA was extracted using TRI reagent (Sigma, USA). A total of 1 μg of RNA was treated with *DNase* I (Fermentas, Canada). First strand cDNA was synthesized using M-MLV reverse transcriptase (Genei, India) as per the manufacturer’s instructions. qPCR was performed using FastStart Essential DNA Green Master (Roche, Germany) in a Light Cycler 96 (Roche, Germany) using perforin specific primers (forward- 5’ GACGCCTACCACAACCT 3’ and reverse 5’ TTTGCCCTCCTAACTGG 3’) designed in Primer Premier Version 5 (Lalitha 2000) from the sequence of contig 113696_50. Briefly, 1 μl of synthesized cDNA was used as a template in a total reaction mixture of 10 μl containing 5 μl of 2X Light cycler SYBR green I mix, 0.5 μl of primer pairs (5 pmole) and 3 μl of H_2_O provided in the kit. The qPCR programme consisted of pre-denaturation at 95°C for 10 min and 45 cycles of amplification at 95°C for 10sec, 55°C for 10sec, and 72°C for 20sec. All reactions were performed simultaneously for each gene with β-actin [9], in the same plate in triplicates. qPCR specificity was verified by melt curve analysis at a temperature of 95°C for 10 s, 65°C for 1 min and 95°C for 1 min. “No-template controls” were included in each run.

The quantification cycle (Cq) values were calculated using a Light Cycler 96 SW 1.1 and the data was exported. N-fold differential expression was calculated using the comparative Cq method [67]. The Cq value of the gene for each cDNA was subtracted from its respective Cq value of β-actin to give a ∆Cq value. Since the samples for each time period were taken in triplicate, an average of the ∆Cq values was obtained. Further, ΔΔCq was calculated by subtracting ∆Cq for post-infection samples from the ∆Cq value of the calibrator (0 h control). Fold difference was calculated as 2^-ΔΔCq^. Mean fold differences and standard errors were calculated. Differences between the temporal mean values of one gene in an organ was analysed using one-way ANOVA followed by Duncan’s multiple range tests, with values *P* < 0.05 considered as significantly different.

### Availability of supporting data

All assembled transcriptome reads are available through GEO Series accession number GSE27994 (http://www.ncbi.nlm.nih.gov/geo/query/acc.cgi?acc=GSE27994). Other supporting data (SNP sequence, annotation and correspondence with chromosome regions in the zebra fish) is included in the additional files section.

## Competing interests

The authors declare that they have no competing interests.

## Authors’ contributions

NR, MB, PKS, and KDM designed the research; PKS, KDM, JNS, SD, YM, PD, NR, and MB performed the research; NR and MB contributed new analytic tools; NR, MK, MA and MB analysed data; and NR, MB, MK and PKS wrote the paper. All authors read and approved the final manuscript.

## Supplementary Material

Additional file 1 **Consensus sex averaged transcribed gene linkage map for *Labeo rohita*.** SNP marker names (contig number followed by position in base pairs) are shown to the right of each linkage group while position (in Kosambi cM relative to the upper marker in the group) is shown to the left. Click here for file

Additional file 2 **Sequence and annotation of contigs containing mapped SNPs (MS-excel file).** LG, linkage group. cM, position on linkage group in centimorgans. GeneID, closest SNP homology from BLAST. snpA and snpB, alternative alleles for SNP. SequenomNot, contig sequence showing alternative SNP allele in square brackets. Click here for file

Additional file 3 **Correspondence of annotated SNPs mapped in *L. rohita* to peptides and chromosome regions in the zebra fish (*Danio rerio*) genome (MS-excel file).** Click here for file

Additional file 4: Figure S1 Frequency of hour’s survival after challenge with *A. hydrophila* within *L. rohita* families A-U. Click here for file
