# Supplementary figures and images for: A linkage map of transcribed single nucleotide polymorphisms in rohu (Labeo rohita) and QTL associated with resistance to Aeromonas hydrophila
Source: BMC Genomics. 2014 Jun 30;15:541. doi: 10.1186/1471-2164-15-541 (PMC4226992; doi:10.1186/1471-2164-15-541)

## LG1

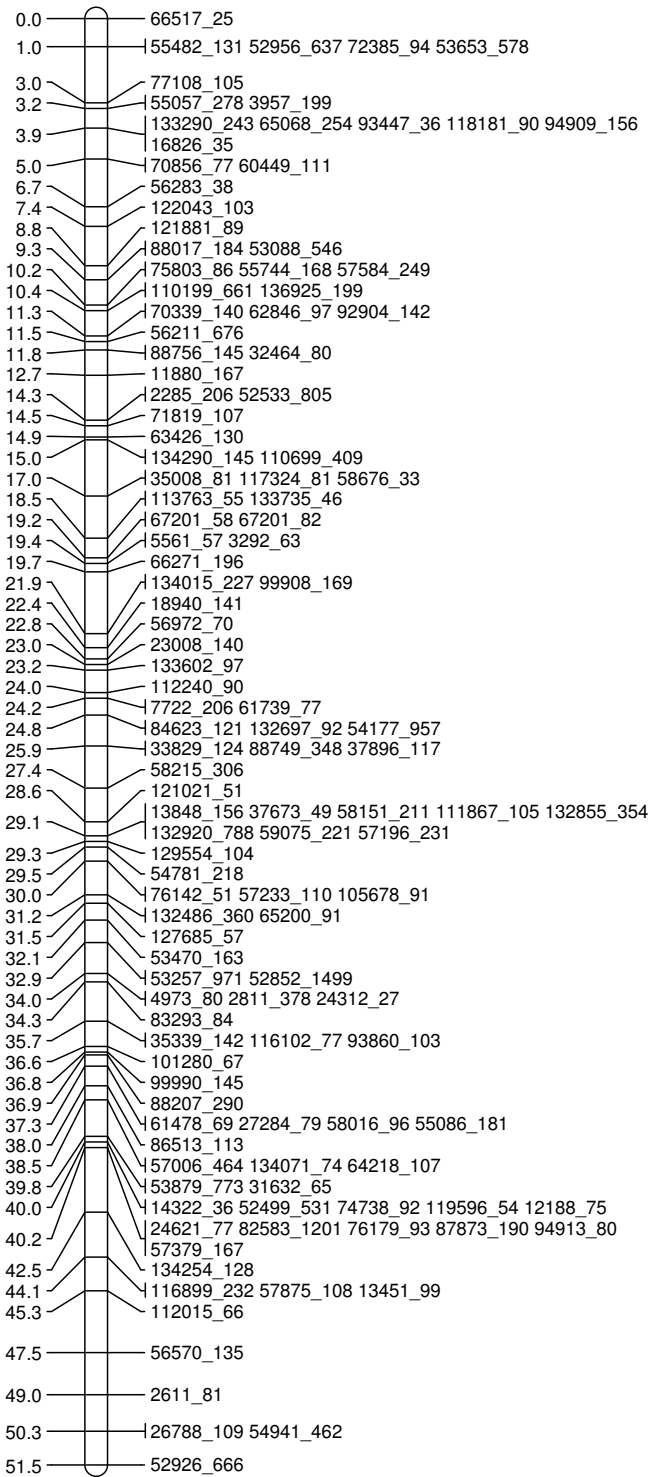

## LG2

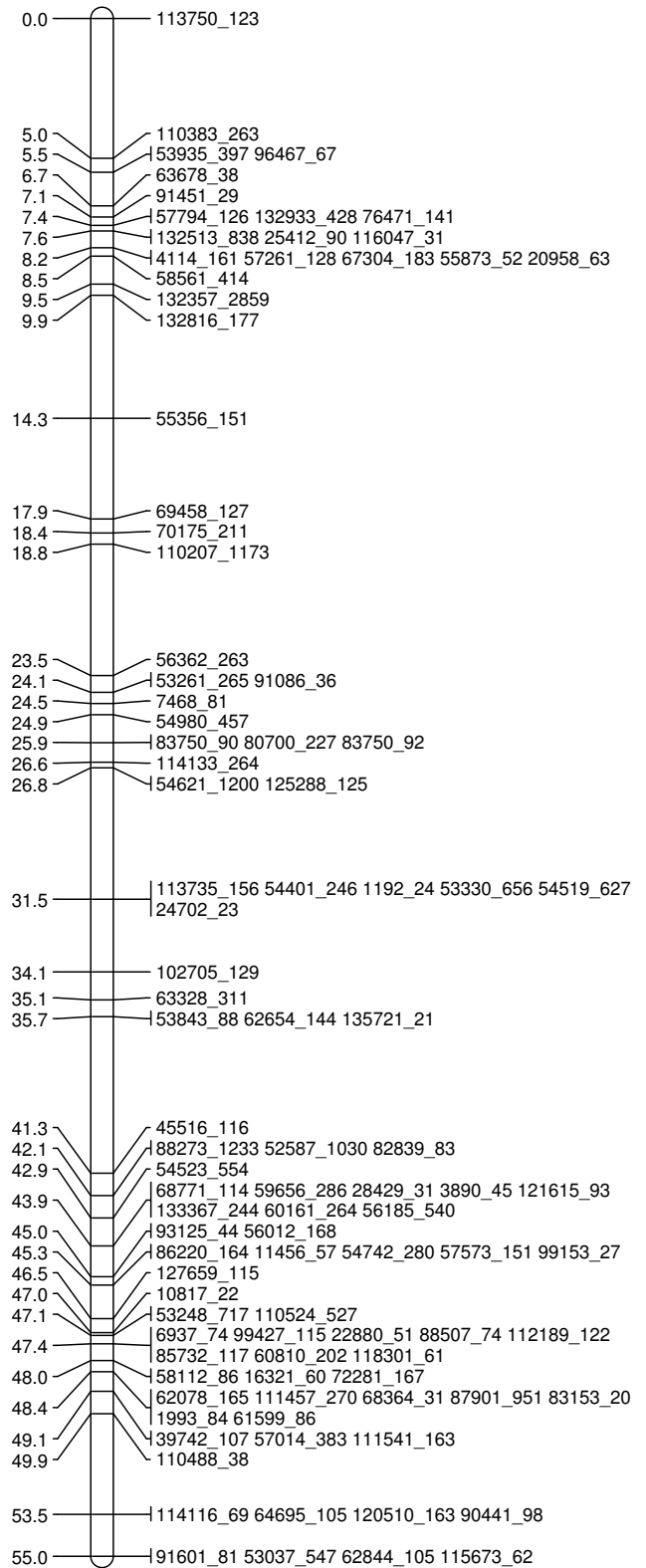

## LG3

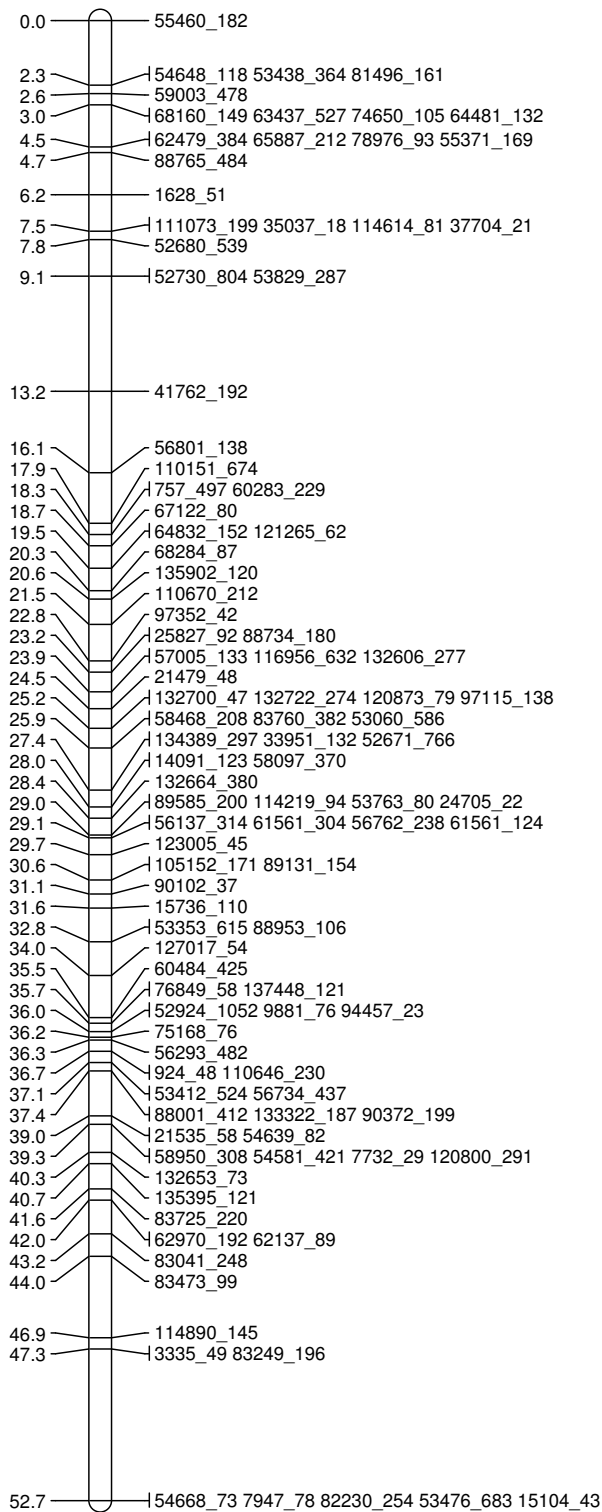

## LG4

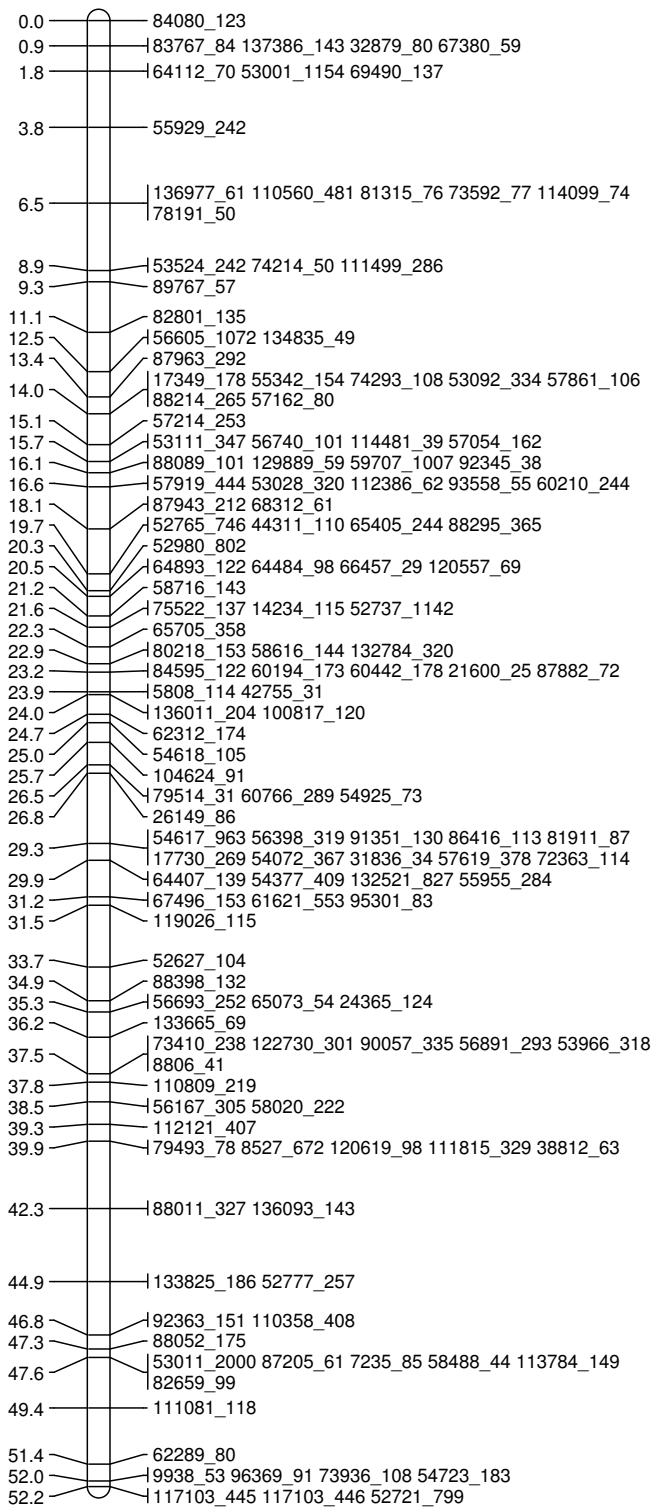

## LG5

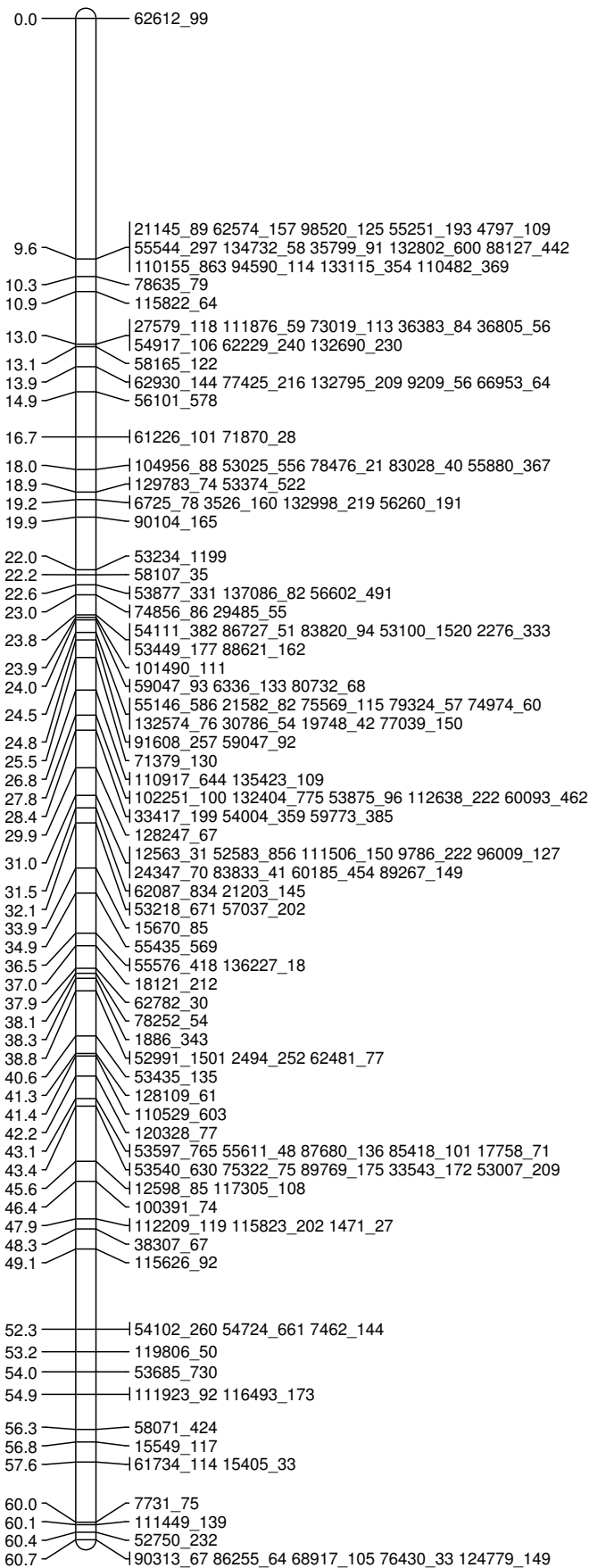

## LG6

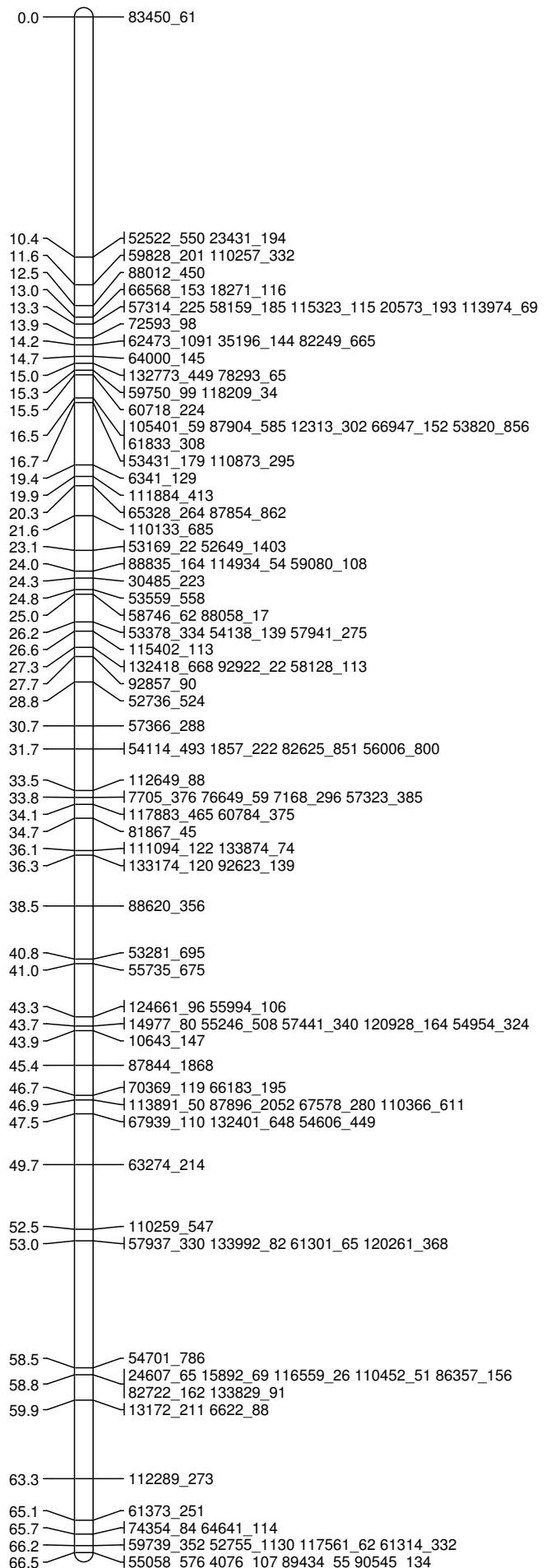

## LG7

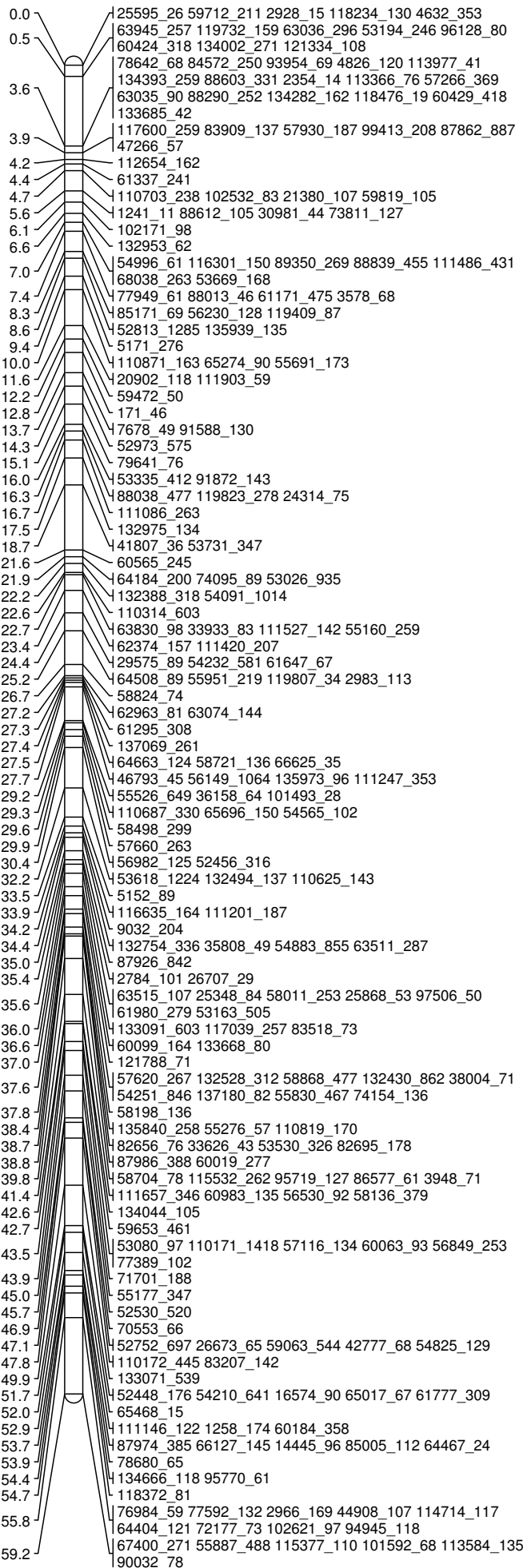

## LG8

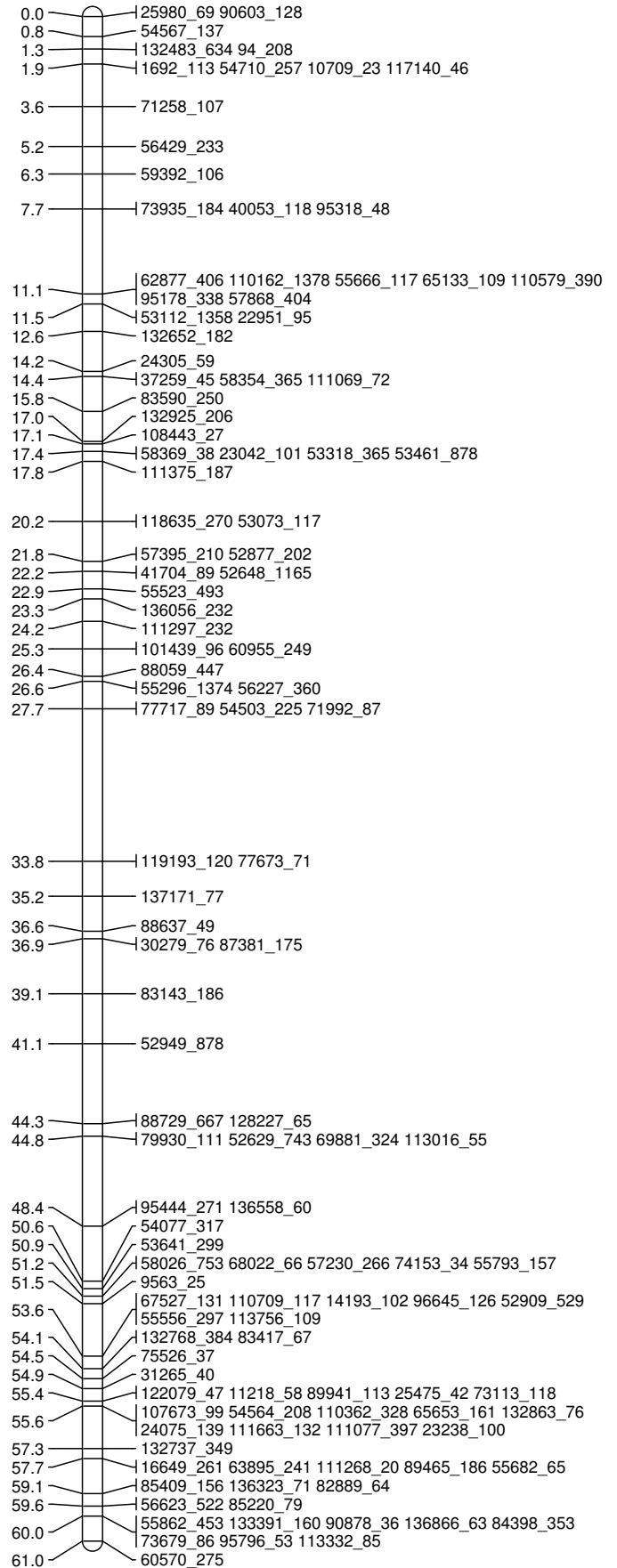

## LG9

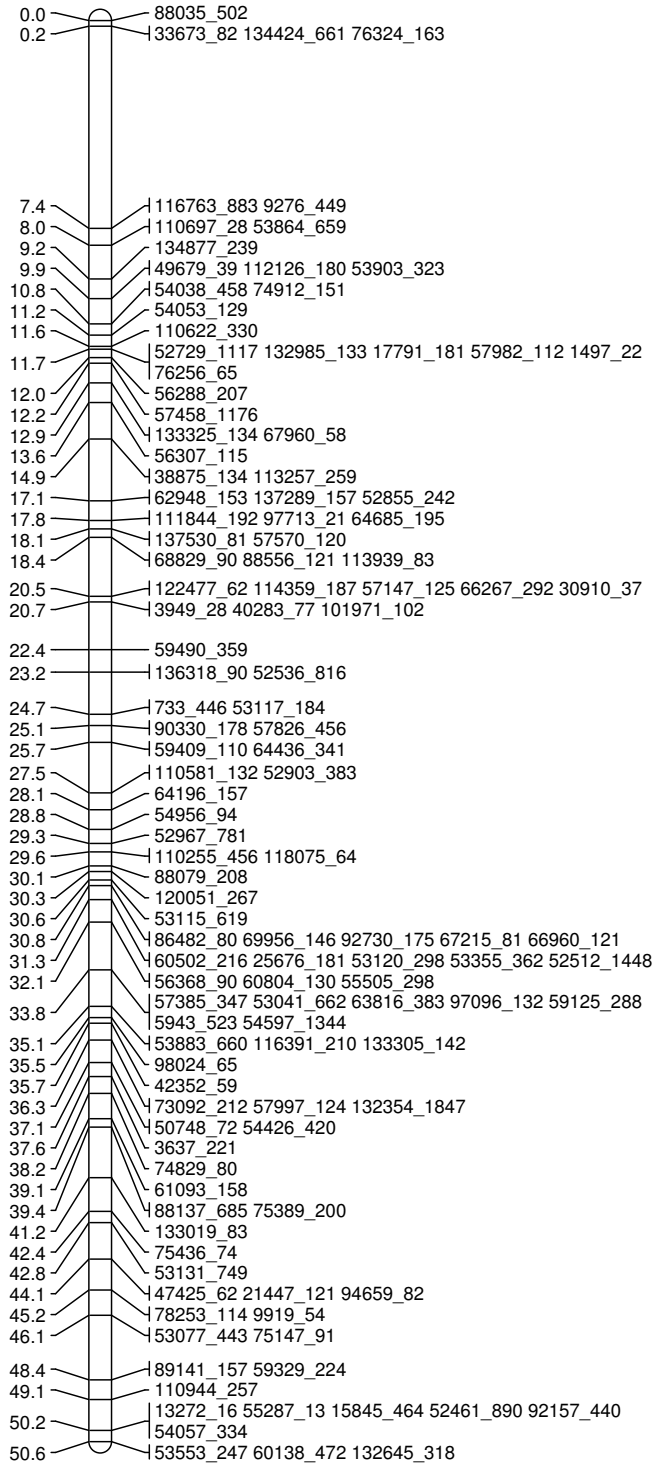

## LG10

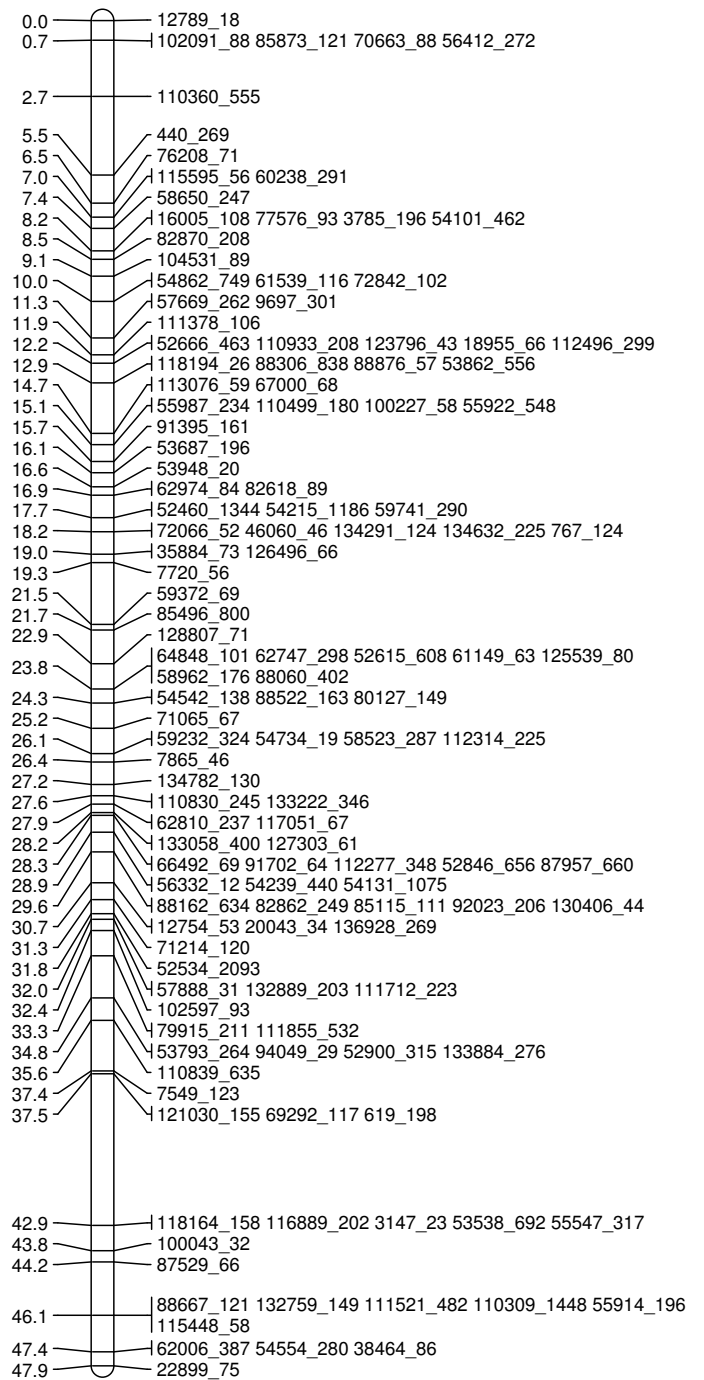

# LG11

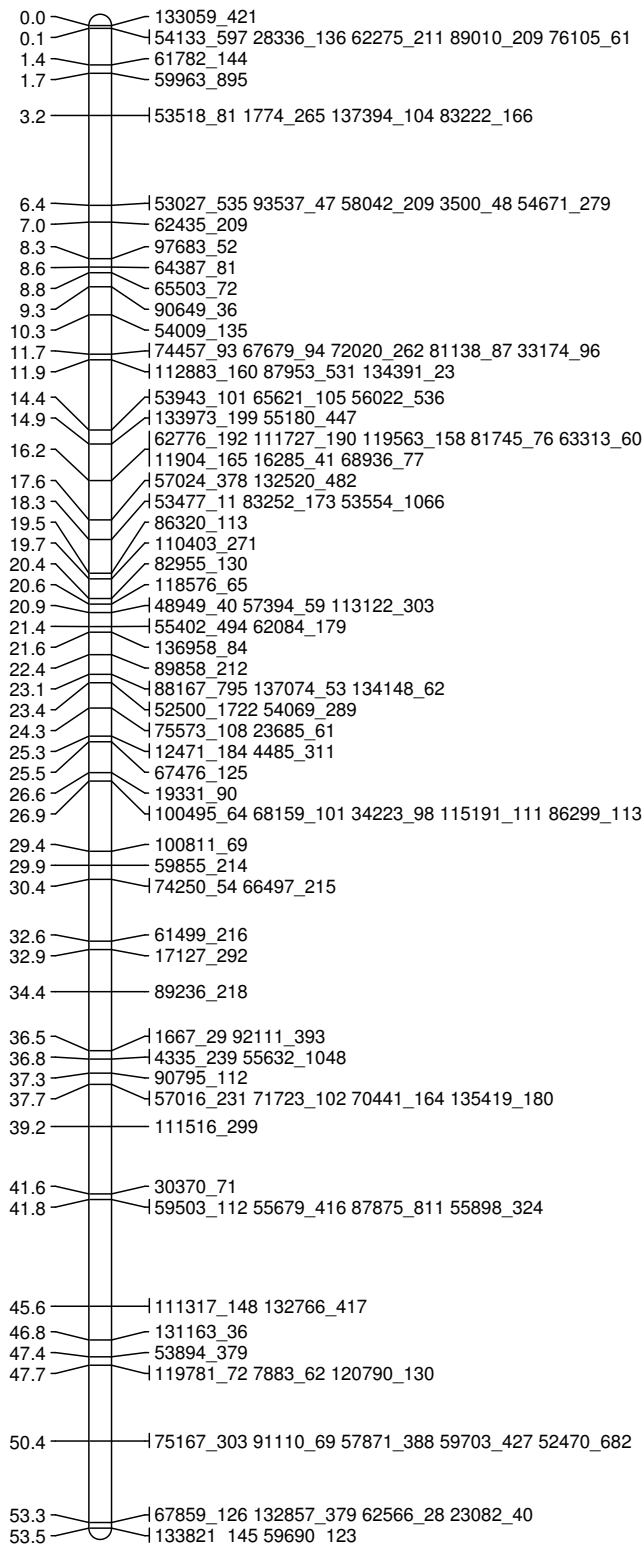

# LG12

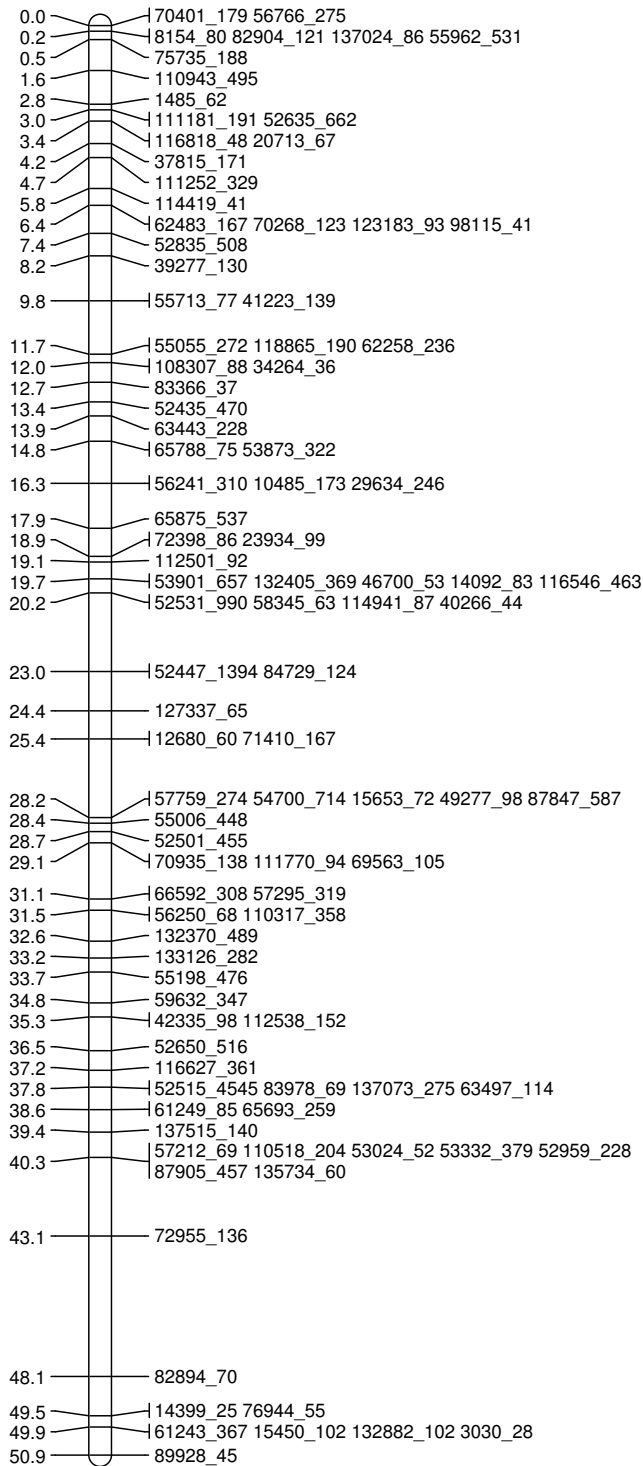

LG13

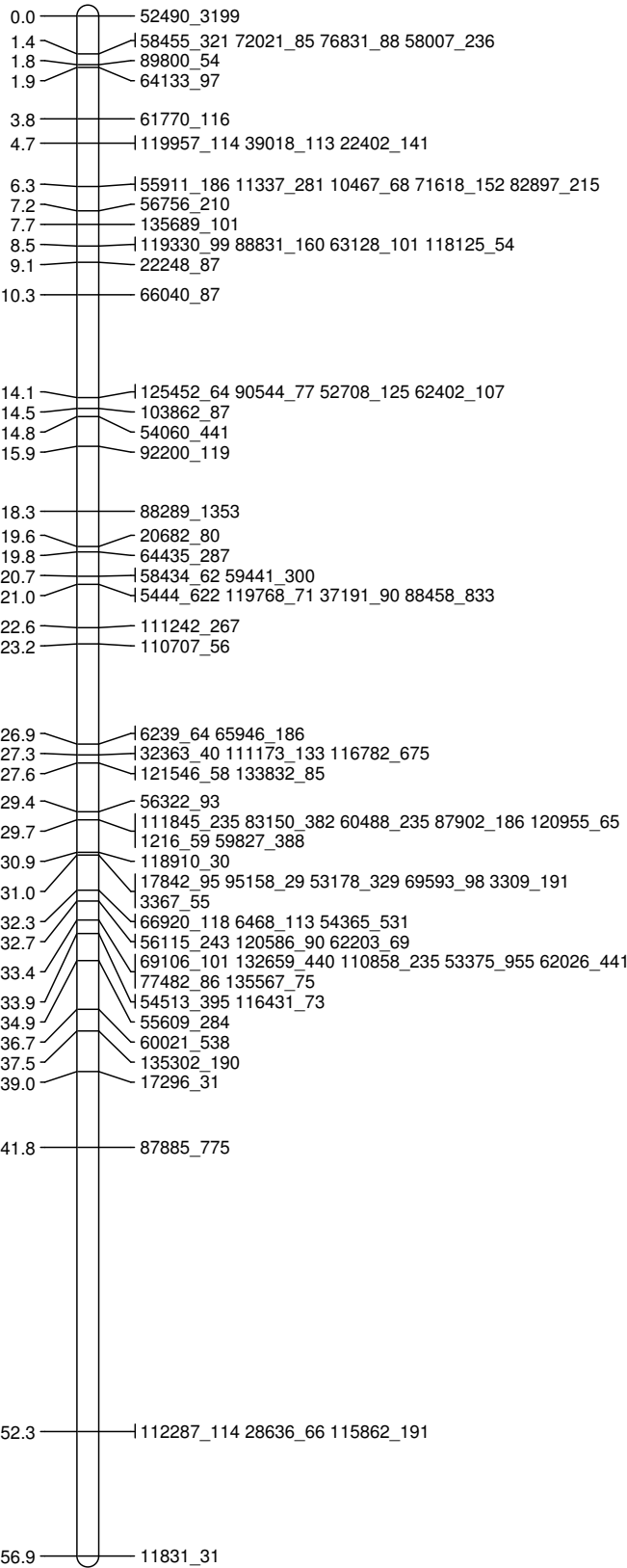

LG14

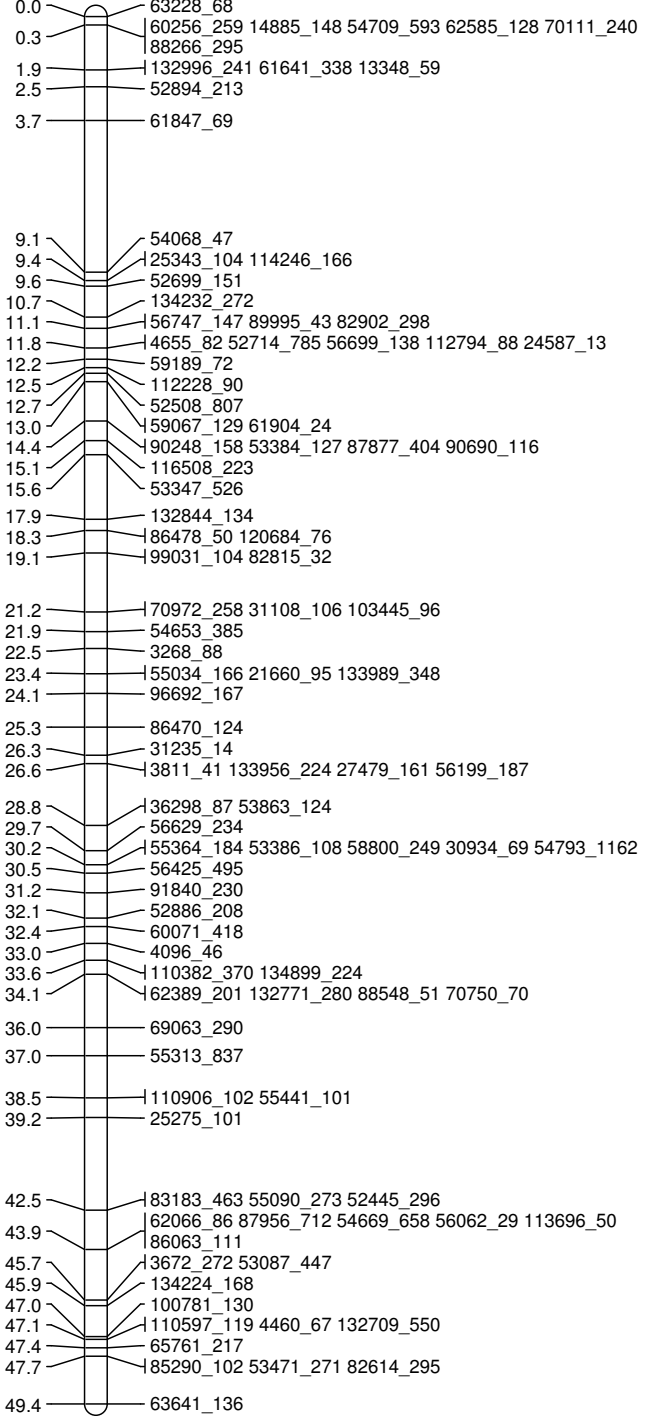

## LG15

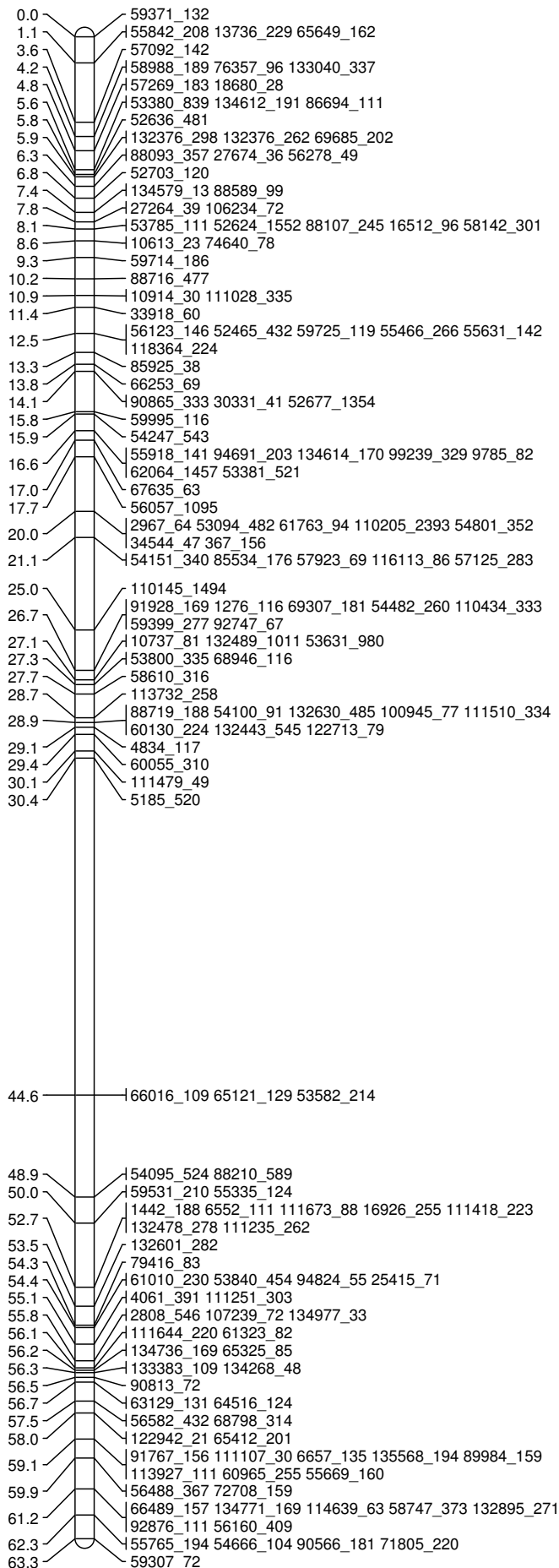

## LG16

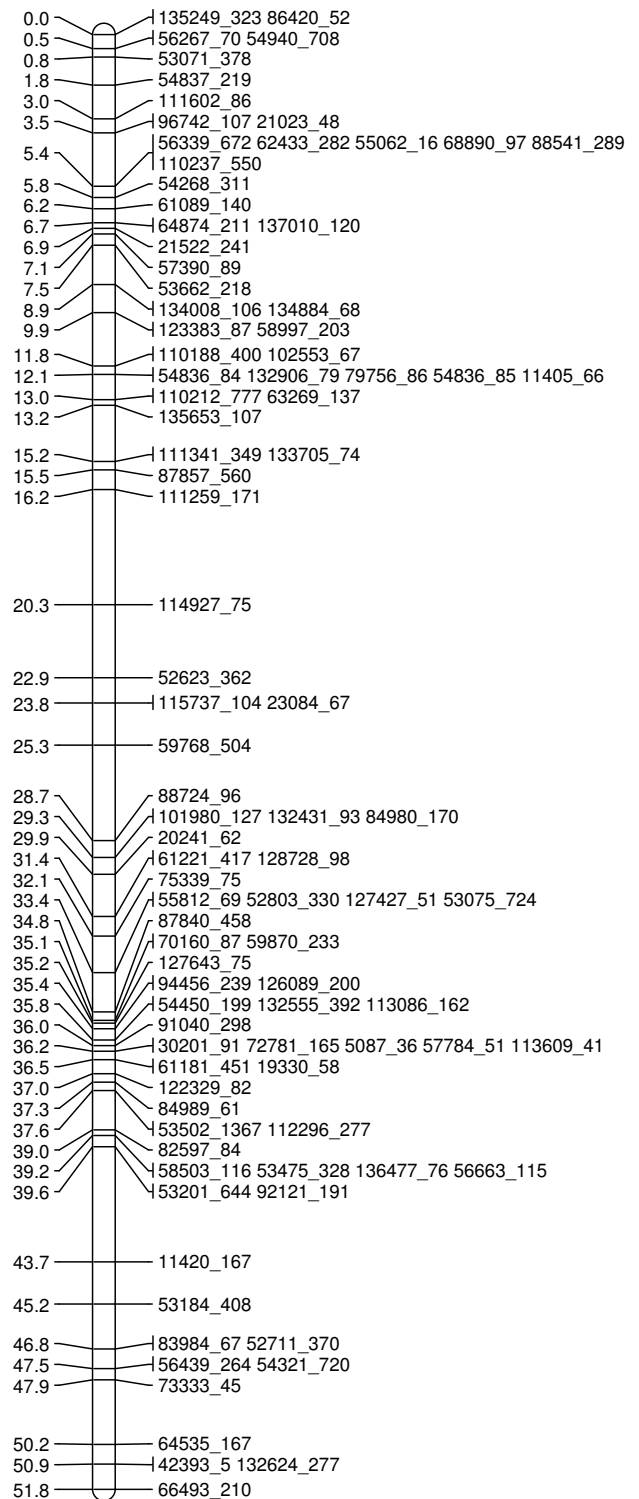

## LG17

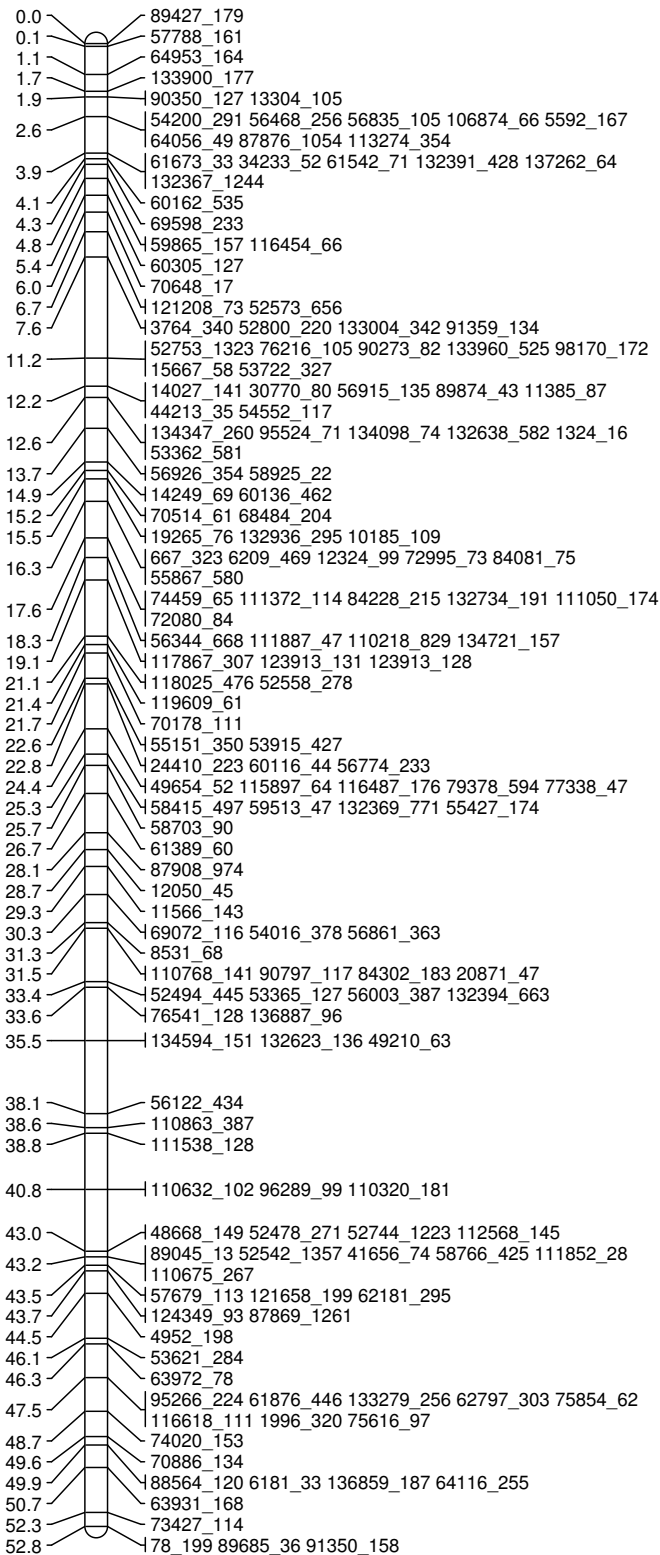

## LG18

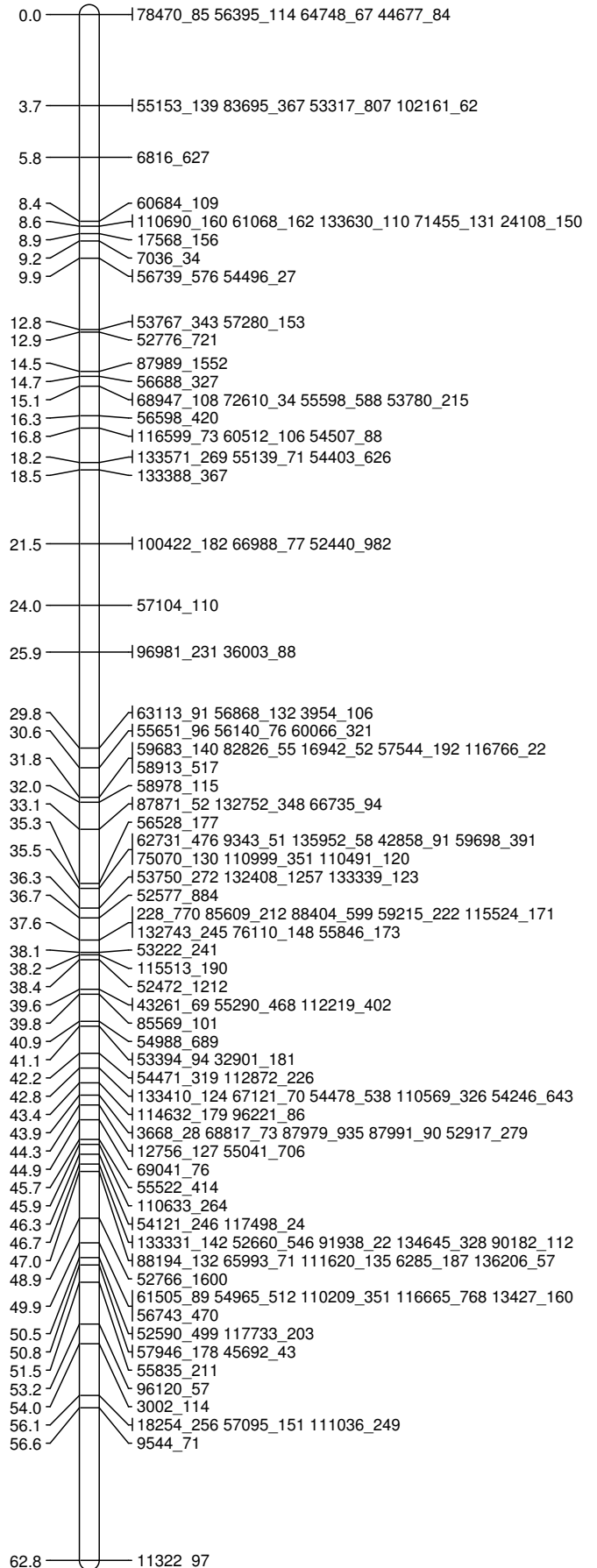

## LG19

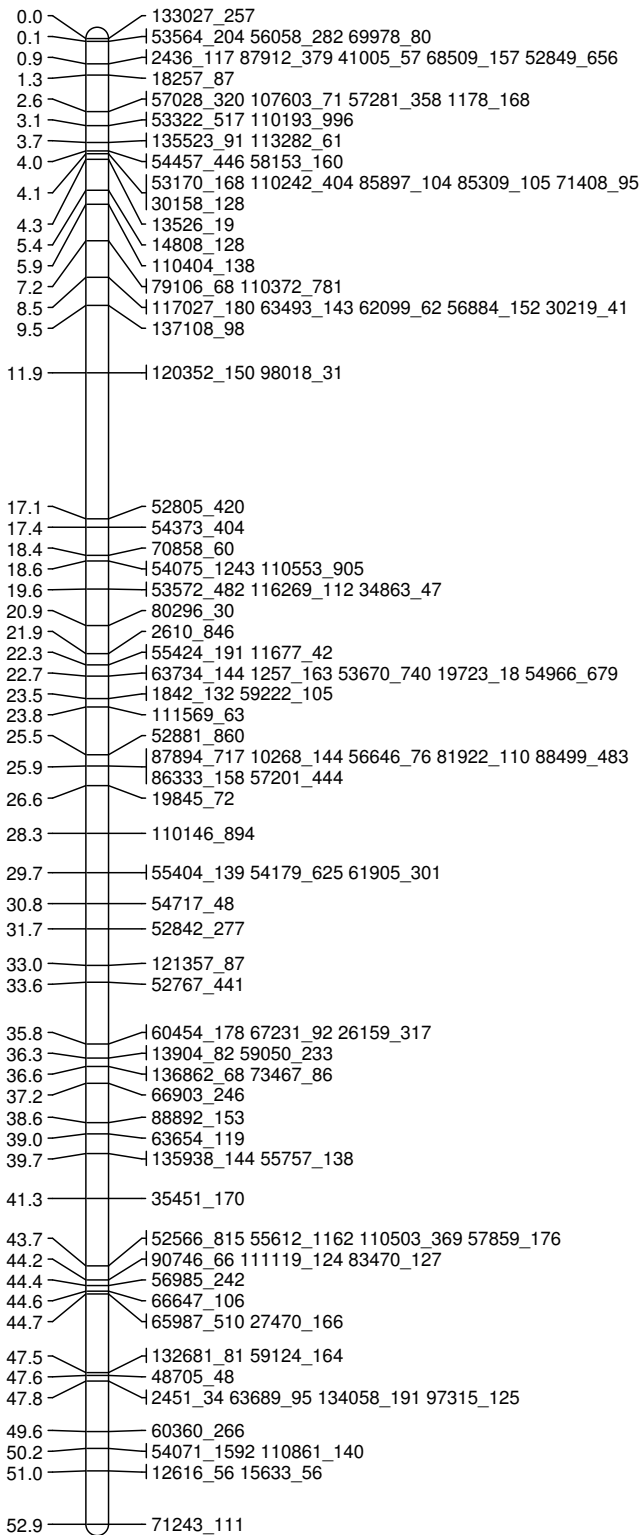

## LG20

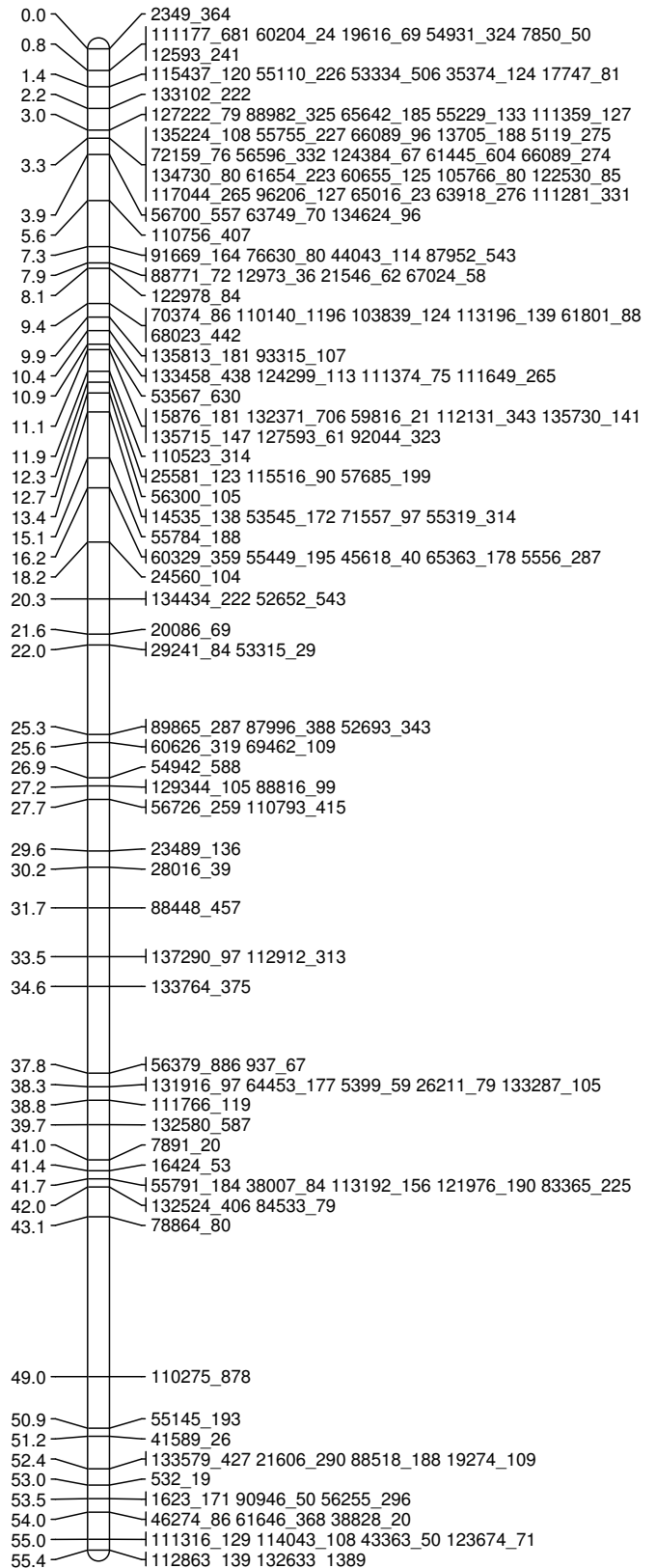

## LG21

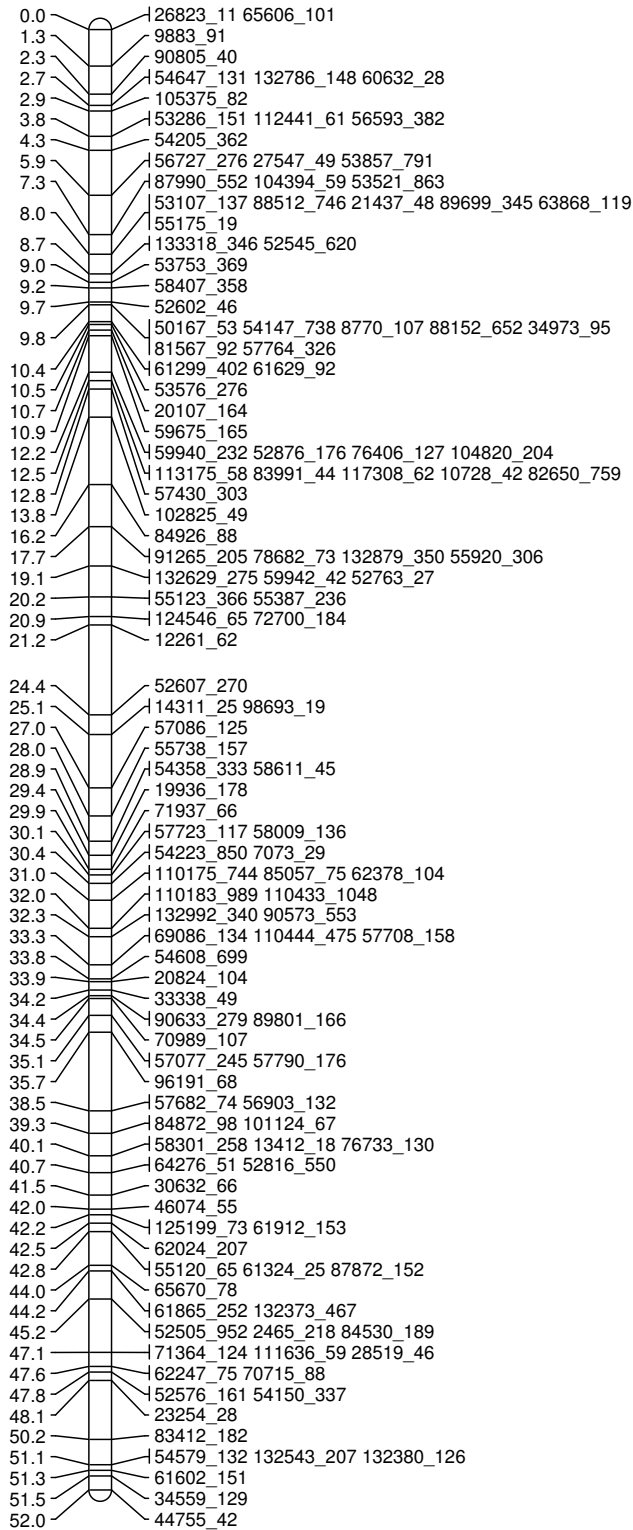

## LG22

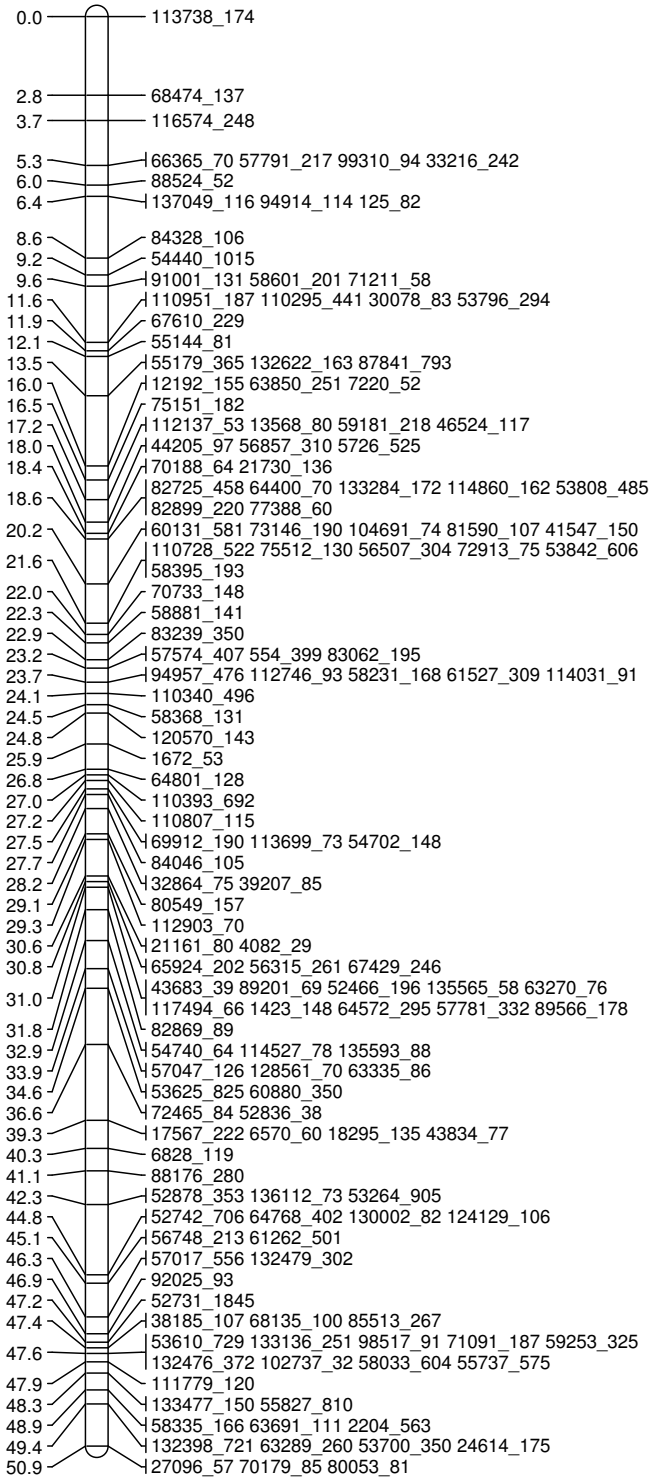

## LG23

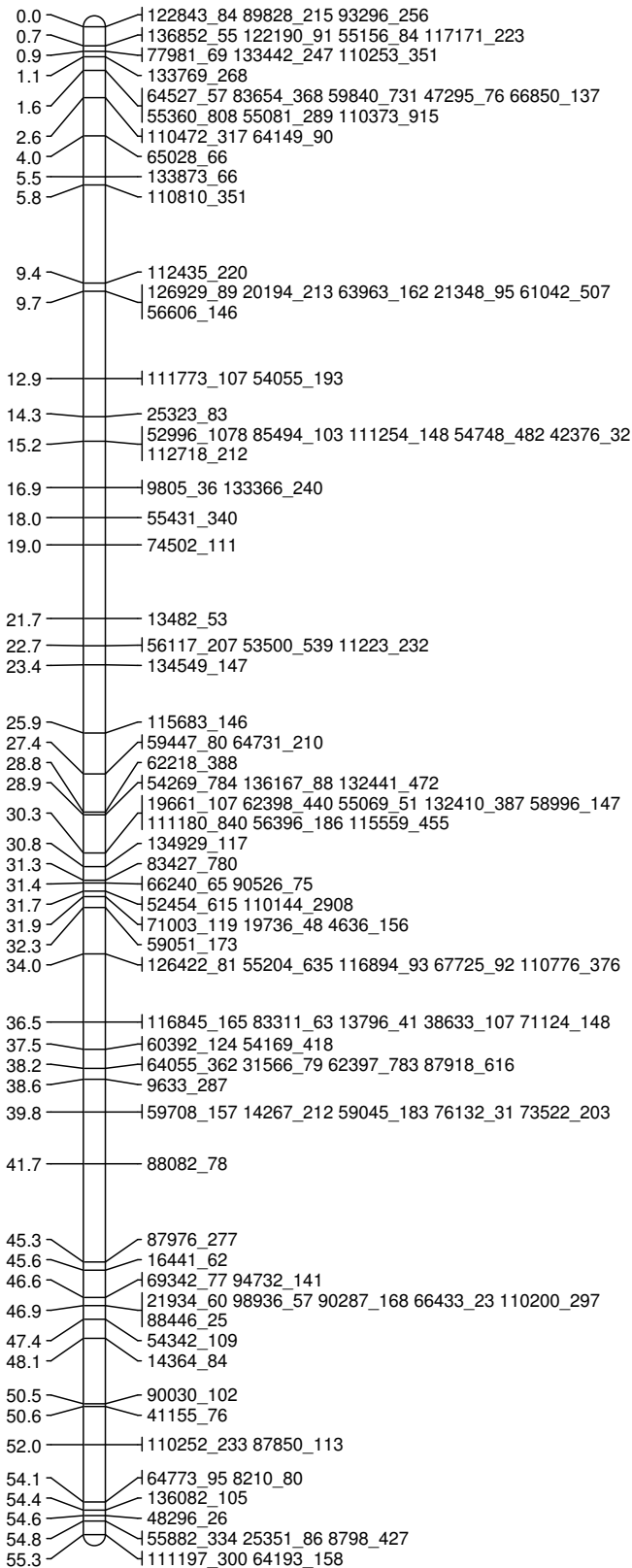

## LG24

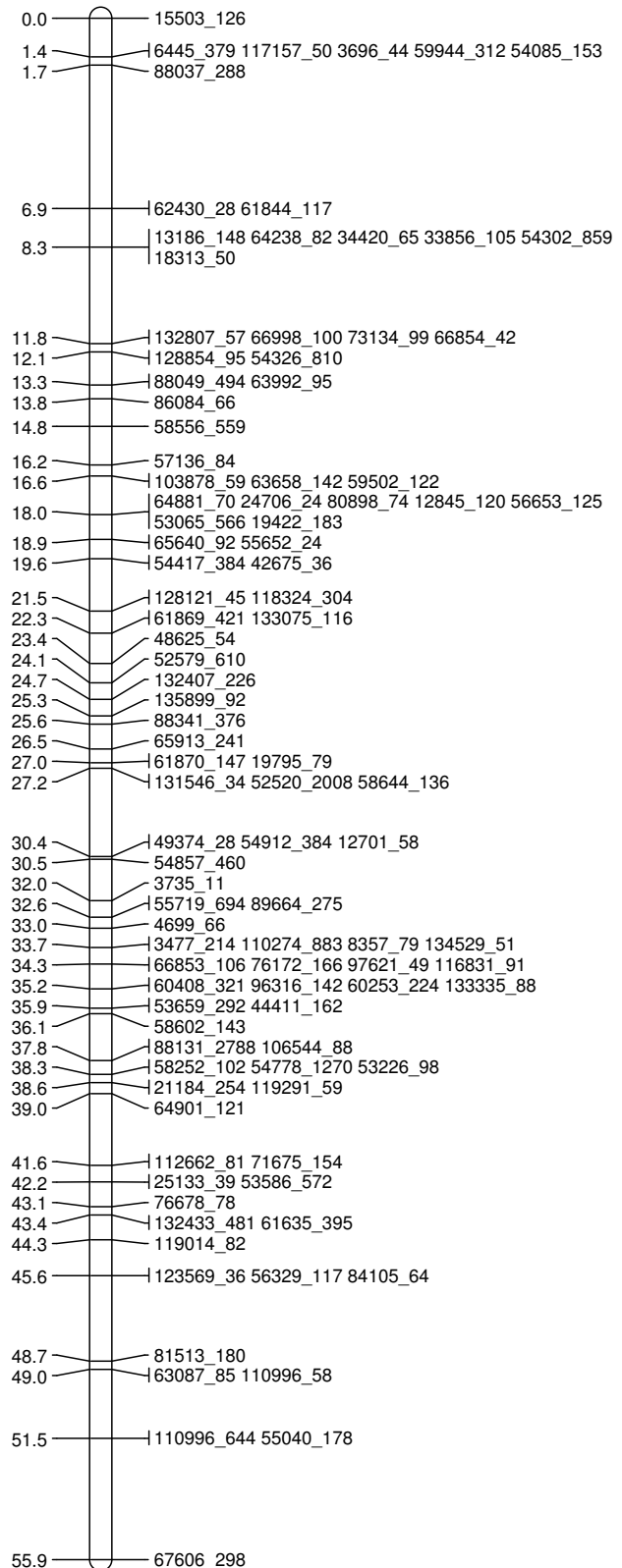

# LG25

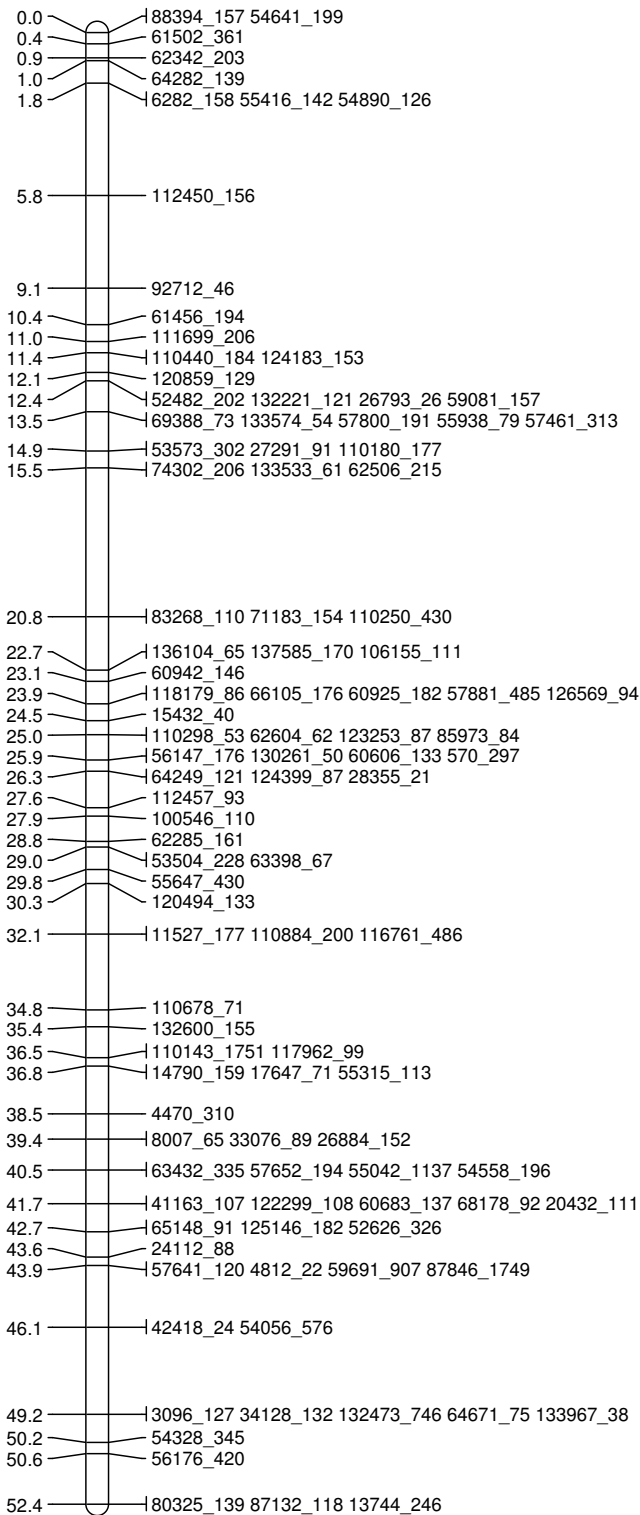

Supplement: Additional file 1 — Consensus sex averaged transcribed gene linkage map for Labeo rohita. SNP marker names (contig number followed by position in base pairs) are shown to the right of each linkage group while position (in Kosambi cM relative to the upper marker in the group) is shown to the left. [file 1471-2164-15-541-S1.pdf]

**A**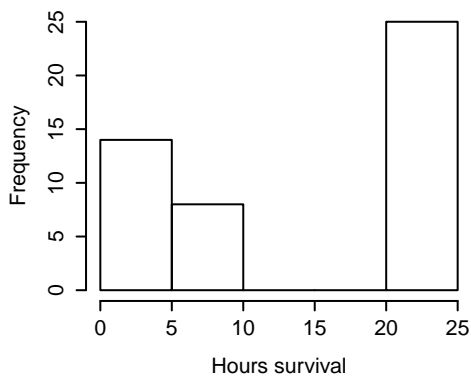**B**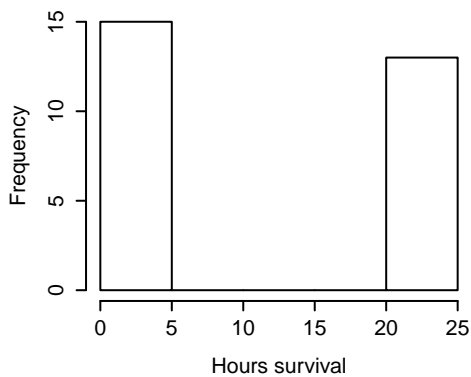**C**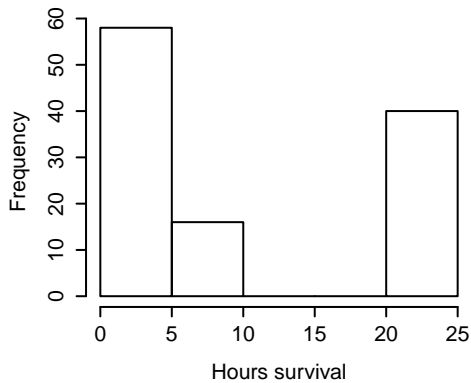**D**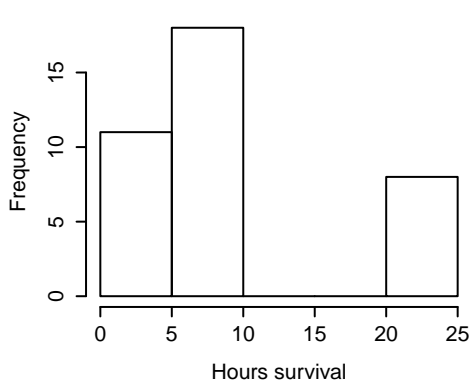**E**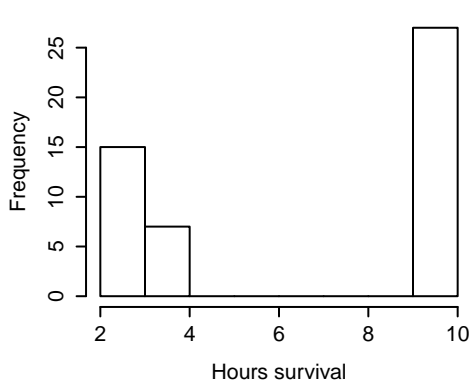**F**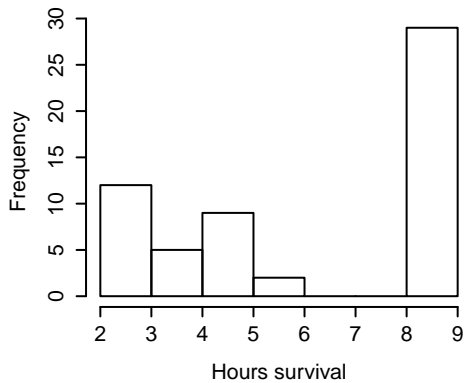**G**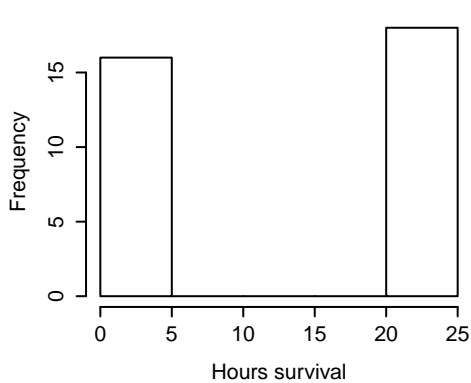**H**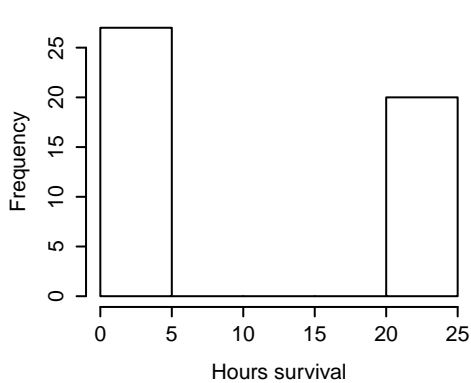

**I**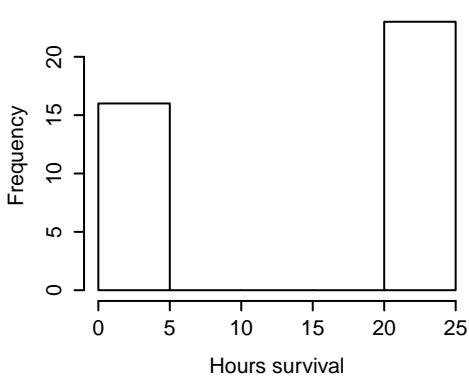**J**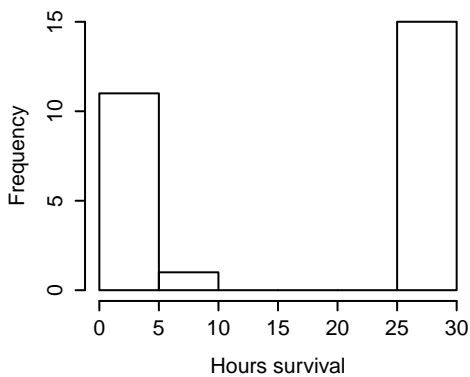**K**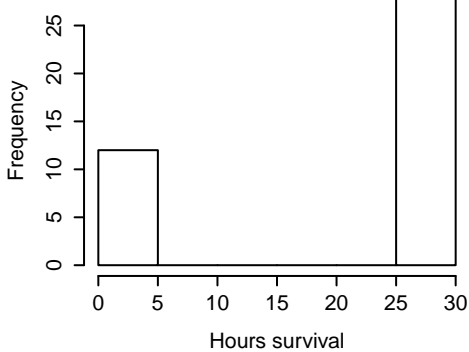**L**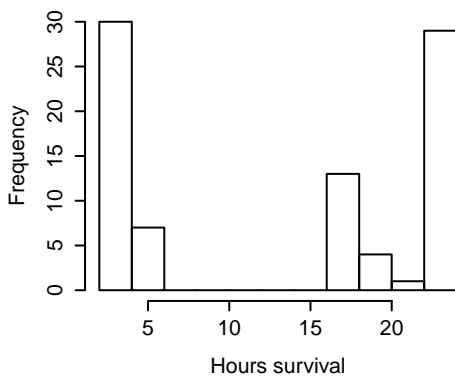**M**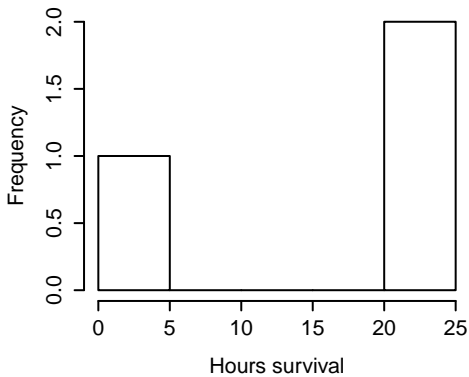**N**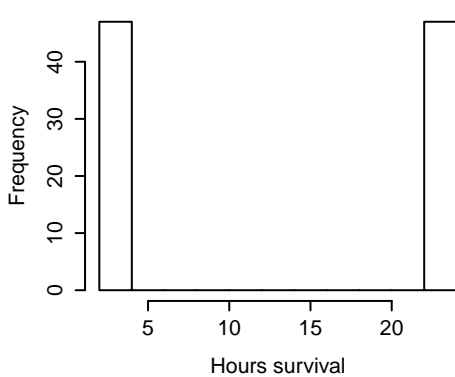**O**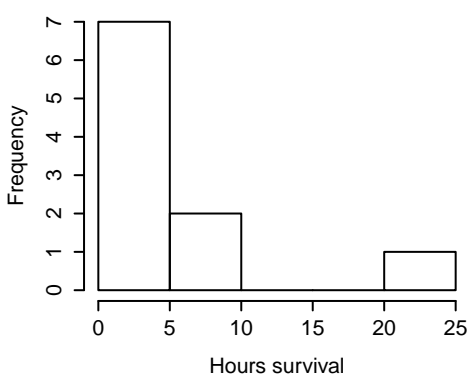**P**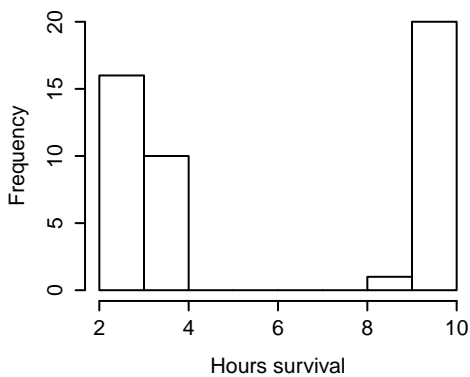

**Q**

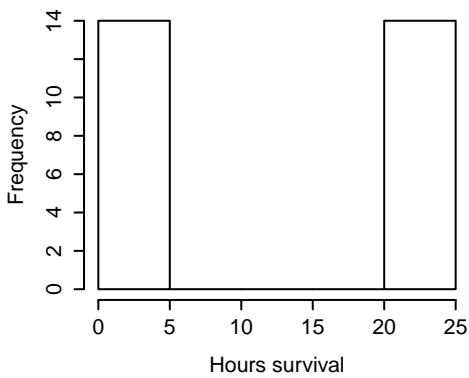

**R**

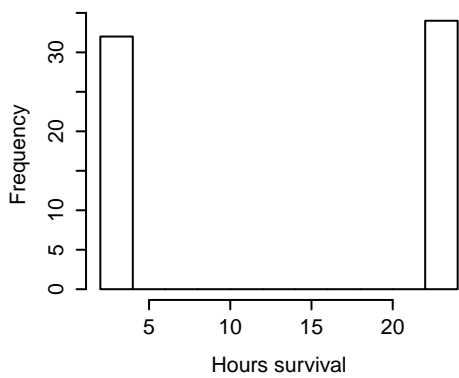

**S**

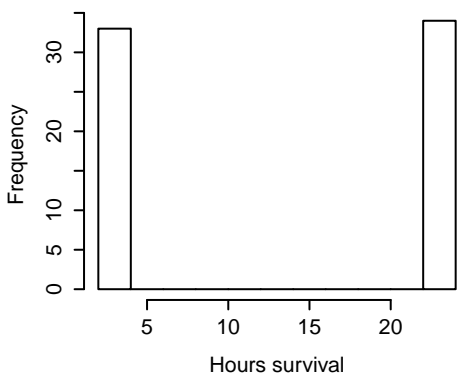

**T**

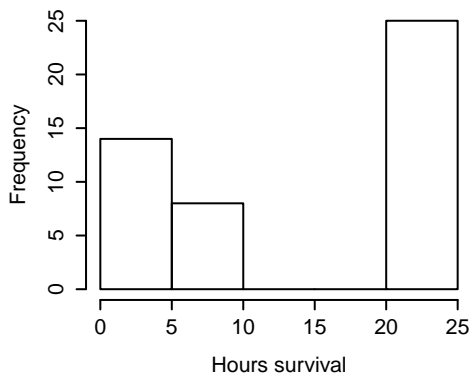

**U**

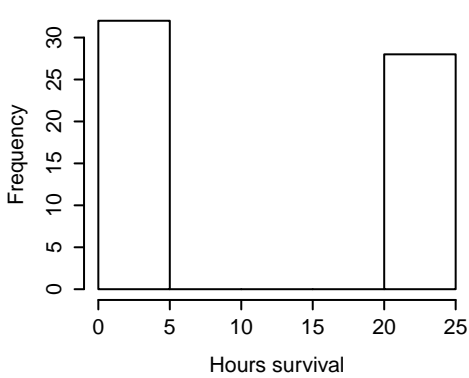

Supplement: Additional file 4: Figure S1 — Frequency of hour’s survival after challenge with A. hydrophila within L. rohita families A-U. [file 1471-2164-15-541-S4.pdf]
